# Gene co-expression analysis of tomato seed maturation reveals tissue-specific regulatory networks and hubs associated with the acquisition of desiccation tolerance and seed vigour

**DOI:** 10.1186/s12870-021-02889-8

**Published:** 2021-03-01

**Authors:** Elise Bizouerne, Julia Buitink, Benoît Ly Vu, Joseph Ly Vu, Eddi Esteban, Asher Pasha, Nicholas Provart, Jérôme Verdier, Olivier Leprince

**Affiliations:** 1 Institut Agro, Univ Angers, INRAE, IRHS, SFR 4207 QuaSaV, 49000 Angers, France; 2 Department of Cell and Systems Biology / Centre for the Analysis of Genome Evolution and Function, University of Toronto, Toronto, ON M5S 3B2 Canada

**Keywords:** Dormancy, Embryo, Endosperm, Longevity, Maturation, Transcriptome, Seed coat, Seed development

## Abstract

**Background:**

During maturation seeds acquire several physiological traits to enable them to survive drying and disseminate the species. Few studies have addressed the regulatory networks controlling acquisition of these traits at the tissue level particularly in endospermic seeds such as tomato, which matures in a fully hydrated environment and does not undergo maturation drying. Using temporal RNA-seq analyses of the different seed tissues during maturation, gene network and trait-based correlations were used to explore the transcriptome signatures associated with desiccation tolerance, longevity, germination under water stress and dormancy.

**Results:**

During maturation, 15,173 differentially expressed genes were detected, forming a gene network representing 21 expression modules, with 3 being specific to seed coat and embryo and 5 to the endosperm. A gene-trait significance measure identified a common gene module between endosperm and embryo associated with desiccation tolerance and conserved with non-endospermic seeds. In addition to genes involved in protection such LEA and HSP and ABA response, the module included antioxidant and repair genes. Dormancy was released concomitantly with the increase in longevity throughout fruit ripening until 14 days after the red fruit stage. This was paralleled by an increase in *SlDOG1–2* and *PROCERA* transcripts. The progressive increase in seed vigour was captured by three gene modules, one in common between embryo and endosperm and two tissue-specific. The common module was enriched with genes associated with mRNA processing in chloroplast and mitochondria (including penta- and tetratricopeptide repeat-containing proteins) and post-transcriptional regulation, as well several flowering genes. The embryo-specific module contained homologues of *ABI4* and *CHOTTO1* as hub genes associated with seed vigour, whereas the endosperm-specific module revealed a diverse set of processes that were related to genome stability, defence against pathogens and ABA/GA response genes.

**Conclusion:**

The spatio-temporal co-expression atlas of tomato seed maturation will serve as a valuable resource for the in-depth understanding of the dynamics of gene expression associated with the acquisition of seed vigour at the tissue level.

**Supplementary Information:**

The online version contains supplementary material available at 10.1186/s12870-021-02889-8.

## Background

Germination and seedling establishment are the first critical factors leading to crop yield. Seed vigour is an estimate of how successful a seed lot will establish seedlings under a wide range of environmental conditions [1]. The desired characteristics for the vigour of tomato seeds correspond to a high and synchronous germination, absence of dormancy, a uniform establishment of seedlings as well as the absence of abnormal seedlings [2–4]. Also, high seed longevity (i.e. the capacity to remain alive during long period of dry storage) is essential to avoid the progressive loss of seed vigour due to cellular deterioration during storage. Longevity is acquired after the establishment of desiccation tolerance (i.e. the capacity to germinate after fast drying). In commercial practice, seed vigour is difficult to achieve as it is a compilation of several traits determined by complex gene by environment interactions. Over 100 quantitative trait loci (QTL) of seed vigour have been identified in a recombinant inbred line (RIL) population originating from a cross between *Solanum lycopersicum* and the wild species *Solanum pimpinellifolium* [2, 3], showing that seed vigour is a complex multifactorial trait.

Seed development is divided in three developmental phases: first, histo-differentiation leads to the formation of the embryo, the endosperm and the seed coat [5–8]. The second phase includes a seed filling phase, characterized by the synthesis of storage reserves followed by a late maturation phase whose duration varies greatly between species [9]. During seed maturation, seed vigour traits are progressively and sequentially acquired [1, 9]. In tomato, germination and desiccation tolerance are acquired during seed filling and fruit expansion, shortly before detachment of the seed from the pericarp [6, 10]. Subsequently, during the next 35–40 days of maturation, seeds acquire their dormancy and resistance against deterioration at high moisture and temperature [6, 11]. Depending on growth conditions, the dormancy acquired during tomato seed development may be partially released at the end of seed maturation [12, 13]. In commercial practice, vigour is considered maximal when the fruit reaches a red and firm stage [11, 13]. Despite its economic importance, we have very little information about the potential longevity of cultivated *Solanum* ssp. seeds.

Information on the regulatory network governing seed maturation and acquisition of seed vigour traits has mostly been described in species such as Arabidopsis, *Medicago truncatula* [14, 15] and soybean [16, 17]. Master regulators controlling seed maturation have been identified in these species, such as LAFL genes namely *LEC1*, *ABI3*, *FUS3*, and *LEC2* [18, 19]. Characterization of their regulatory networks revealed their role in seed storage and induction of desiccation tolerance together with dormancy, longevity and degreening among vigour traits (reviewed in Leprince et al. 2017 [9]). Additional analysis of coexpression networks based on “guilt by association” in maturating seeds of *M. truncatula* and Arabidopsis has also revealed ABI3-independent pathways and additional transcription factors that are associated with seed longevity, such as ABI5, WRKY3 and NFXL1 [14]. Characteristically, in these species the fruit and seed undergo maturation drying, resulting in a loss of cellular water to below 10%, formation of the cytoplasm into solid-like state, and a state of quiescence in the seed tissues. In contrast, tomato seed maturation occurs entirely in a hydrated environment that maintains the seed moisture around 30–50% [5, 12]. Such high water content allows metabolic activities to carry on until fruit harvest and seed drying. During development, precocious germination within the fruit tissue is prevented by the negative osmotic potential of the environment of the seed, the presence of abscisic acid (ABA), and the mechanical strength of the seed coat and endosperm [12, 20, 21].

Apart from the role of ABA and gibberellins (GA) in regulating vivipary, the regulatory pathways leading to acquisition of longevity and dormancy (and possibly release) during development of tomato seeds remain unknown. It was shown that *procera* mutant seeds that do not exhibit any DELLA activity germinated much faster than wild types and died very rapidly during dry storage [22], indicating the implication of the GA signalling pathway. Another important regulator of dormancy and longevity is DELAY OF GERMINATION 1 (DOG1), whose protein levels accumulate during seed maturation and correlate with the depth of dormancy [23, 24]. In Arabidopsis, DOG1 integrates environmental cues, such as temperature, via ABA and ethylene signalling pathways [25]. In tomato, *DOG1* transcript levels increase throughout maturation [26] but how they correlate with dormancy remains to be assessed.

In tomato, the endosperm remains preponderant throughout seed maturation. Together with the maternal seed coat tissue, the endosperm is also important for seed vigour. During seed development, it plays an important role in supporting embryonic growth by supplying nutrients, protecting the embryo and controlling embryo growth by acting as a mechanical barrier [27]. During seed development in rice, endosperm also acts on seed dormancy and germination by affecting ABA signalling via sugar metabolism [28]. In tomato, the endosperm cap weakening during imbibition that results from the hydrolysis of the galactomannans within the cell walls constitutes a pre-requisite to ensure the completion of radicle emergence ([29] and references therein). After germination, storage reserves that accumulated in the endosperm are important to sustain growth during seedling emergence [3]. Moreover, across the Angiosperms, the endosperm is thought to exert a negative effect on seed longevity as endospermic seeds exhibit on average a 3-fold lower life span during dry storage than non-endospermic seeds [30, 31]. To date, genome-wide transcriptional profiling studies to identify important regulators and processes required for endosperm development have mainly focussed on cereals where most of the endosperm tissues undergo programmed cell death and drying at the end of seed development [32, 33]. In contrast, the endosperm of tomato remains alive throughout seed development.

Besides the role of the endosperm, genetic evidence indicates that the acquisition of seed vigour is also regulated via a molecular dialogue between the embryo and the seed coat [34, 35]. For example, the seed coat was found to control the development of the embryo through the synthesis of 12-oxo-phytodienoic acid (OPDA), an oxylipin occurring nearly exclusively in these tissues that represses germination [35]. While transcriptome data on whole seeds are available for tomato [26], understanding how seed vigour is acquired in tomato at the molecular level requires the study of these seed tissues individually during maturation.

Here, we identify the gene modules associated with the acquisition of seed vigour during tomato fruit ripening at the tissue level. The acquisition of vigour traits was characterized through 14 developmental stages from 15 to 90 days after flowering (DAF), including fruit over-ripening. Tissue-specific RNA-seq data were generated, and a weighted gene co-expression network analysis (WGCNA) was carried out to identify temporal and tissue-specific gene modules. Using a trait-based measure of significance, gene modules and hubs were identified that are linked to the acquisition of desiccation tolerance, longevity, dormancy release and germination under osmotic stress.

## Results

### Physiological characterization of tomato seed maturation

**Fig. 1:**
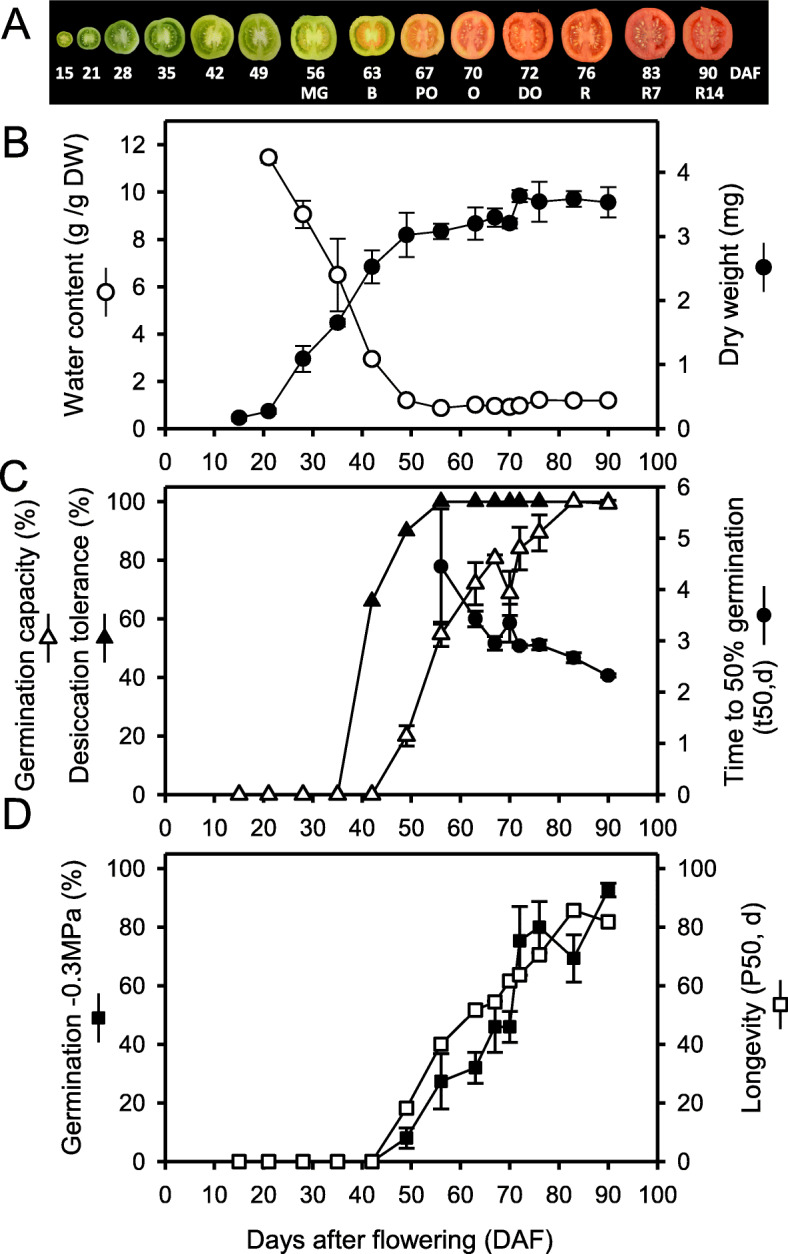
Physiological characterisation of tomato seed maturation. **a.** Time course of tomato fruit development and correspondence between seed age (days after flowering), fruit colour and fruit developmental stages (MG, mature green; B, breaker; PO, pale orange; O, orange; DO, dark orange; R, red; R7, red plus 7 days of ripening; R14, red plus 14 days of ripening). **b.** Changes in seed water content and dry weight (DW) during seed development. Data are the mean (± SD) of three biological replicates. **c.** Changes in desiccation tolerance (germination in the presence of 30 mM KNO_3_ after fast drying), germination capacity in water (germination without 30 mM KNO_3_ after fast drying) and speed of germination. Data are the average of three replicates (± SD) of 50 seeds. **d.** Acquisition of the ability to germinate under stressful conditions (− 0.3 MPa) and seed longevity (P50)

Different physiological traits acquired during seed maturation were characterized based both on seed age calculated after tagging the flowers (i.e. 15–90 DAF) and on the fruit ripening stages (i.e. from mature green (MG) to over-ripe fruit (red fruit + 14 d)) as shown in Fig. 1a. Seed filling occurred between 21 to 49 DAF as observed by the increase in seed dry weight (DW) and decrease in water content (Fig. 1b). During seed filling, desiccation tolerance (i.e. the ability to germinate after fast drying) was acquired from 35 DAF onwards, at the start of endosperm solidification, until 56 DAF, consistent with previous studies [5, 6]. Thereafter, seed water content remained high at 1 g H_2_O/g DW (50% fresh weight basis) throughout fruit ripening, highlighting that the developing tomato seeds do not undergo a maturation drying while they remain in the fruit. Germination percentage of artificially dried seeds progressively increased from 42 DAF until 83 DAF, corresponding to over-ripened fruits (Fig. 1c). This increase in germination was attributed to a gradual release of primary dormancy. Indeed, 100% of the dried immature seeds from the mature green stage onwards (56 DAF) germinated when they were directly imbibed in 30 mM KNO_3_, a treatment known to break dormancy [36]. Furthermore, germination speed (time to reach 50% germination, t50) increased from 4.4 d at 56 DAF to 2.3 d at 90 DAF (Fig. 1c, Additional file 1: Fig. S1). From 42 DAF onwards, seeds gradually acquired their ability to germinate at low water potential (− 0.3 MPa). In parallel, longevity (measured as P50, the period required for the seed batch to lose 50% of germination during storage, Additional file 1: Fig. S2) also increased progressively, even after the fruit red stage, considered to be optimal for seed quality (Fig. 1d). Altogether, these data pinpoint a long late maturation phase lasting over 40 days from 42 DAF onwards during which seed vigour is progressively increased.

### Spatial and temporal description of the tomato seed transcriptome

**Fig. 2:**
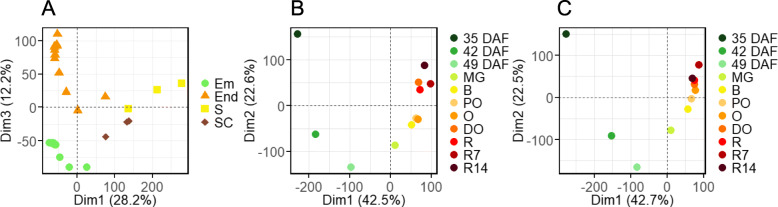
Principal component analysis of developmental transcriptome of tomato seeds. **a.** Relatedness of indicated the seed tissues (Em, embryo; End, endosperm; S whole seeds; SC, seed coat) throughout the development. **b.-c.** Developmental time-series of embryo **b** and endosperm **c**

To obtain a spatial and temporal representation of the seed transcriptome during development, a RNA sequencing (RNA-seq) data series of whole seeds and isolated seed tissues was obtained at 14 stages throughout seed development from 15 to 28 DAF for entire seeds (S), from 35 DAF to stage R14 for embryo (Em) and endosperm (End), and from 35 to 49 DAF for seed coat (SC). Principal component analysis (PCA) was performed to compare changes between the transcriptomes of the different seed tissues and developmental stages (Fig. 2). This revealed a distinct clustering of transcript profiles corresponding to tissue types throughout development (Fig. 2a). PCA carried out on embryo and endosperm respectively (Fig. 2b and c) indicated that the major factor responsible for the variance of both datasets (Dim1, 43% of the variation explained) was aligned to the developmental stages in chronological order between 35 DAF until 49 DAF. A major transcriptional switch occurred between 49 DAF and mature green fruits. Thereafter, the variation was partially explained by the second dimension, which aligned transcriptome changes with the progress of fruit ripening.

**Fig. 3:**
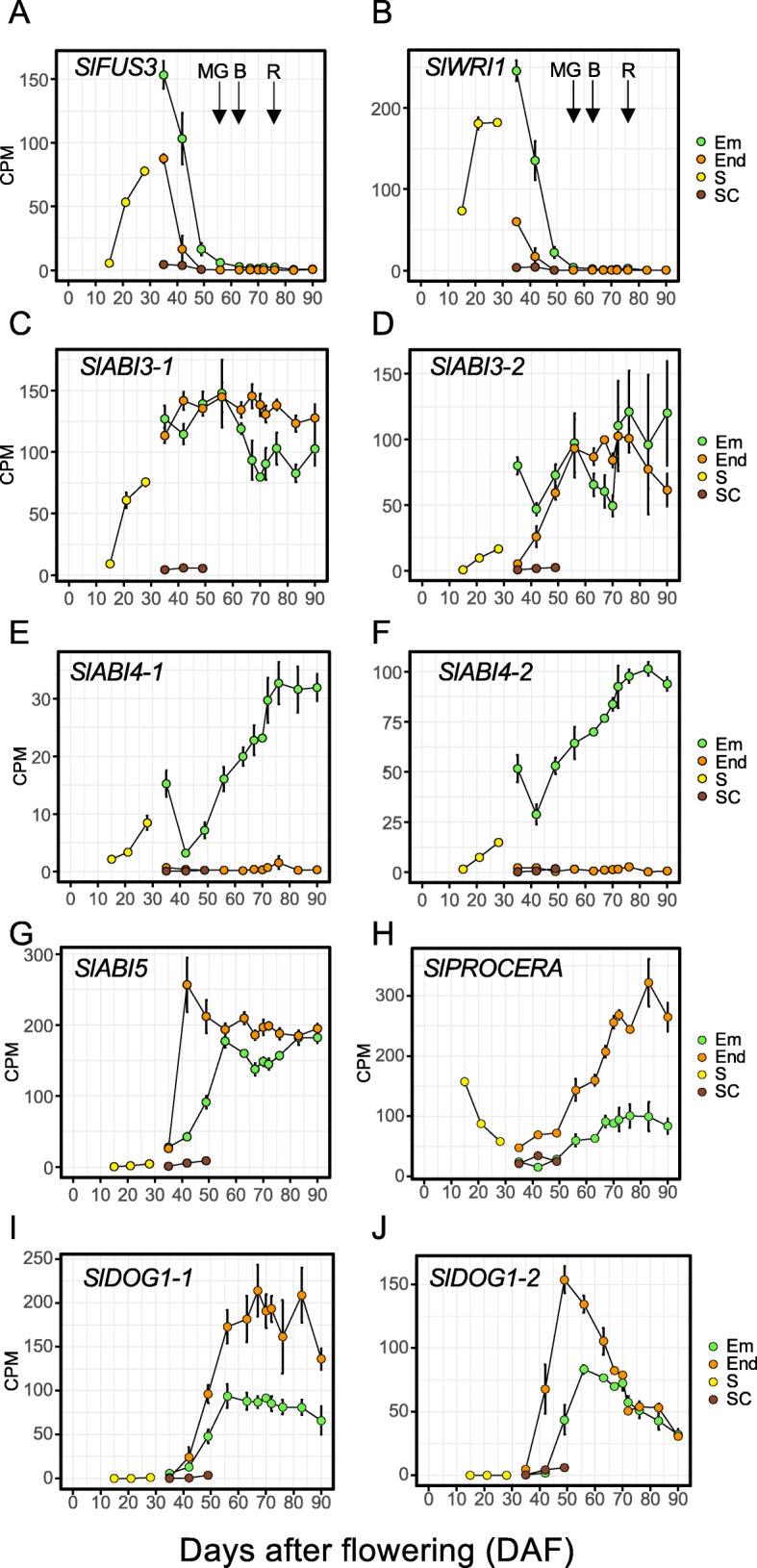
Expression profiles of key regulatory genes during seed development. **a.** Sl*FUS3*, *Solyc02g094460.2.*1; **b.** Sl*WRI1*, *Solyc01g096860.3.1*; **c** Sl*ABI3–1*, *Solyc06g083600.3.1*; **d.** Sl*ABI3–2,* Solyc06g083590.4.1; **e.** Sl*ABI4–1,* Solyc03g095973.1.1; **f.** *ABI4–2, Solyc03g095977.1.*1; **g.** Sl*ABI5, Solyc09g009490.4.*1, ***H.*** *PROCERA, Solyc11g011260.1.1*; **i.** *DOG1–1, Solyc02g072570.2.*1; **j.** *DOG1–2*, *Solyc03g006120.4.1*. Arrows indicate the following fruit maturity stages, MG, mature green; B, breaker, R, red. Data are the means (± SD) of three biological replicates. Em, embryo; End, endosperm, S, whole seed, SC, seed coat

The timing of molecular events during tomato seed development was further examined via the expression of major regulatory genes involved in embryogenesis, seed filling and seed vigour acquisition (Fig. 3, Additional file 1: Fig. S3). Transcript levels of the orthologs of *SlFUSCA3*, one of the four master transcriptional regulators forming the LALF maturation network, increased from 15 DAF onwards and were maximum around 40 DAF (Fig. 3a). Thereafter, transcripts rapidly decreased and were no longer detectable at the mature green stage. As a marker of seed filling, we chose the ortholog of *WRI1*, a target of *LEC2* regulating oil accumulation in Arabidopsis seeds [37]. *SlWRI1* transcript abundance followed a similar pattern as *SlFUSCA3*, being higher in the endosperm compared to embryo (Fig. 3b). Two *ABI3* orthologs were detected in the tomato genome. Transcript levels for *SlABI3–1* increased between 15 DAF and 42 DAF, as for *SlFUSCA*3, whereas *SlABI3–2* transcript levels reached a maximum 20 d later, around 60 DAF, when fruits were mature green (Fig. 3c, d). The expression of both genes preceded the acquisition of desiccation tolerance (Fig. 1c). Thereafter, for both genes, transcript levels remained high throughout the rest of maturation, with a slightly lower expression in the embryo for *SlABI3–1* (Fig. 3c). The other two members of the LAFL network, *LEC1* and *LEC2* could not be identified with enough confidence and were not included in this study. As other representatives of ABA signalling pathway, we selected *ABI4* and *ABI5*, the latter controlling the accumulation of protective molecules involved in seed longevity in legumes [38]. Two orthologs of *ABI4* were identified, and their transcripts were detected specifically in the embryo. Transcript levels increased between 42 to 76 DAF, when fruits became red, and remained high upon further ripening (Fig. 3e, f). *SlABI5* transcript levels increased later than *SlABI3*, but earlier than *SlABI4*, first in the endosperm, with a maximum at 42 DAF, then in the embryo, with a maximum at 56 DAF, when fruits were mature green (Fig. 3g). We also included *DOG1*, a key regulator of seed dormancy that is expressed during seed maturation in Arabidopsis downstream of *LEC1* [39], and *PROCERA*, the only DELLA annotated gene in tomato whose GRAS domain regulates dormancy and longevity [22]. Transcript levels of two *SlDOG1* genes increased from 42 DAF onwards, with higher levels in the embryo compared to endosperm (Fig. 3 i, j, Additional file 1: Fig. S3). Whereas *SlDOG1–1* (*Solyc02g072570.2.1*) remained higher upon further maturation, transcript levels of *SlDOG1–2* (*Solyc03g006120.4.1*) peaked at 49DAF, and decreased progressively upon further maturation, in parallel with the release of dormancy (Fig. 1c). Transcript levels of *SlPROCERA* were high during early development around 15 DAF in whole seeds, consistent with previous data [26]. Thereafter, they decreased to very low levels around 35 DAF, and increased again progressively until 80 DAF (Fig. 3h). Transcript levels of *SlPROCERA* were 3-fold higher in the endosperm compared to the embryo, and followed a similar profile as for *SlABI4*. Both *SlABI4* orthologues and *SlPROCERA* transcripts correlated with the increase in longevity and germination under osmotic stress (compare Fig. 3 with Fig. 1d).

### Identification of temporal and tissue-specific gene modules via WGCNA

**Fig. 4:**
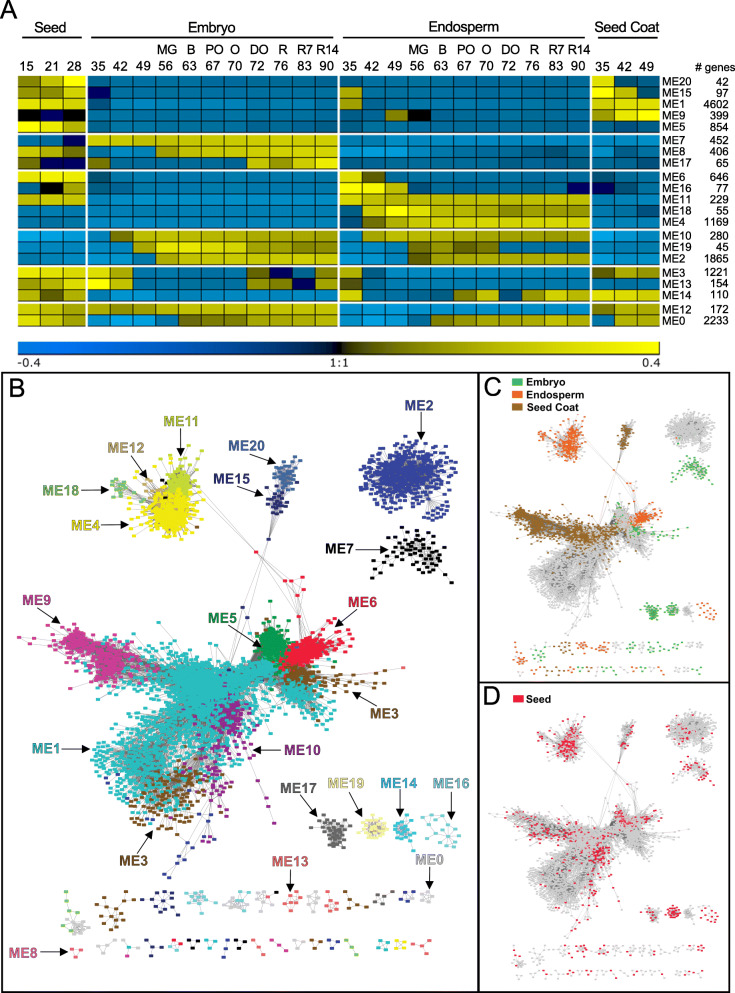
Gene co-expression network of tomato seed development. **a.** Heatmap of module eigengene expression profiles throughout development in whole seeds and indicated seed tissues. Material was harvested at the indicated days after flowering. The corresponding fruit ripening stage is also shown. Module detection is explained in Additional file 1: Fig. S4. **b.-d.** Weighted gene co-expression network visualized using Cytoscape. **b.** Projection of expression modules, each colour corresponding to one module. **c.** Projection of preferentially expressed genes in the embryo (green), endosperm (orange) or seed coat (brown). **d.** Projection of seed preferentially expressed genes (red). ME, module eigengene

To identify and characterize the temporal and tissue-specific gene modules, we carried out a WGCNA [14, 40]. After normalization, genes with low expression value and low coefficient of variation between replicates were discarded, resulting in 15,173 genes that were used for the network analysis. The soft thresholding power to calculate adjacency was based on the criterion of approximate scale-free topology while limiting the loss of mean connectivity [41]. Here, the scale-free topology value was set at 0.8 (Additional file 1: Fig. S4A). Hierarchical clustering analysis revealed 21 distinct expression modules containing between 42 and 4602 genes, which were represented by their module eigengenes (ME, Fig. 4a, Additional file 1: Fig. S4B). The gene modules were sorted in five groups based on their expression pattern and tissue specificity (Fig. 4a). The first group corresponded to genes expressed early during seed development, both in whole seeds and in the seed coat, but not during maturation (ME20, ME15, ME1, ME9 and ME5). The second (ME7, ME8, ME17) and the third group (ME6, ME16, ME11, ME18, ME4) corresponded to modules with genes exhibiting an embryo- and an endosperm- specific expression, respectively characterizing the second half of the seed development (Fig. 4a). The fourth group (ME10, ME19, ME2) was characterized by modules with transcripts expressed both in embryo and endosperm and specific to late maturation. The fifth group (ME3, ME13, ME14) corresponded to modules containing genes expressed first early during seed development and again later during the end of maturation. ME12 was a hybrid module containing genes expressed in the embryo or in the endosperm. ME0 consisted of genes whose expression did not fit any modules.

To visualize the gene modules, the weighted gene co-expression network was examined using Cytoscape [42]. The network contained 6399 nodes and 411,826 edges on which the expression modules were projected in different colours for a topological representation (Fig. 4b). The network was made up of a central core and several sub-networks of variable size, two of them being connected to the central core. To further characterize the network topology, we determined transcripts that were preferentially expressed in endosperm, seed coat or embryo (Additional file 2: Table S1). Preferentially expressed genes were determined as transcripts that were at least 10-fold more abundant in a tissue compared to any other seed tissues. Projection of these genes on the network revealed that embryo preferential transcripts were mostly found in three disconnected subnetworks (ME7, ME17, ME19) and at the edge of ME3 within the central core of the network (Fig. 4b-c). Endosperm preferential transcripts localized mostly in ME6 and in the tightly connected modules ME18, ME11, ME4. The seed coat transcriptome was also well delimited within the network, mostly represented in ME9 and ME1 and in the loosely connected subnetwork within ME15 and M20 (Fig. 4b-c). Seed preferentially expressed genes were identified as transcripts whose abundance was a least 10-fold higher than that of other tomato plant tissues that were extracted from published studies [26, 43, 44]. They were found throughout the gene network (Fig. 4d), but with a high concentration in endosperm- and embryo-specific subnetworks ME4, ME19 and ME10 modules (Fig. 4 b, d).

### Characterization of biological processes in tissue-specific modules

**Fig. 5:**
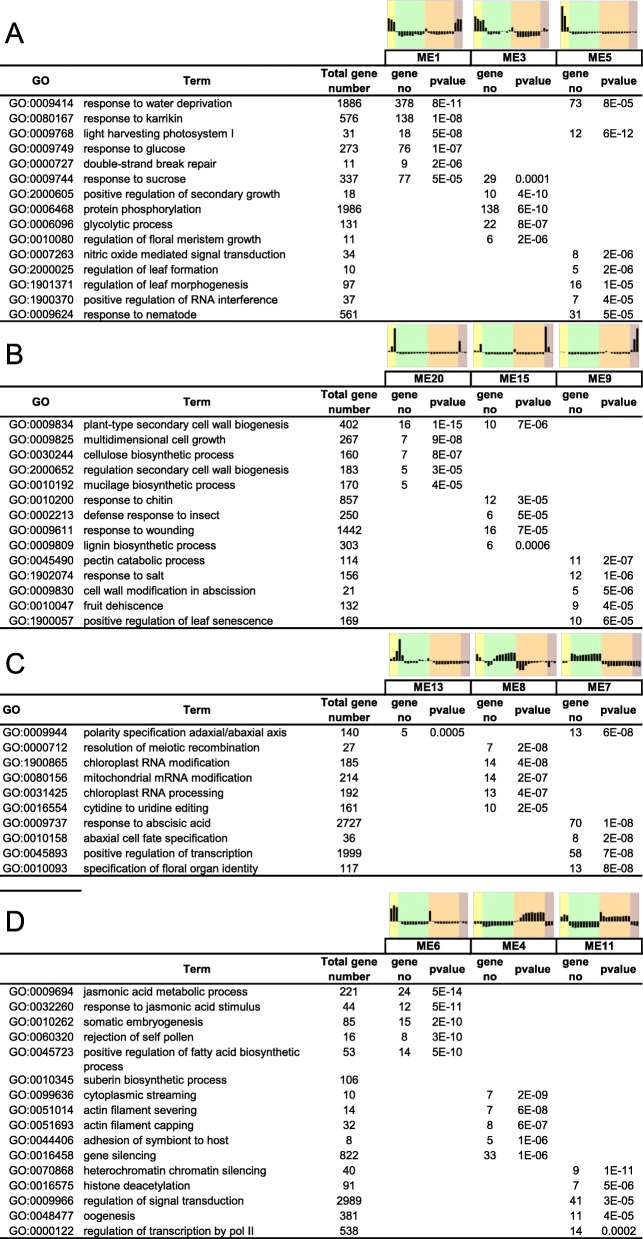
GO term enrichment analysis of general and tissue-specific expression profiles during seed development. **a.** all tissues **b.** seed coat. **c.** embryo. **d.** endosperm. Only the top 5 significantly over-represented GO terms are shown. The eigengene expression profile is shown for each ME as an aid to the eye. The different colours represent the different tissues across developmental stages as shown in Fig. 4a: yellow, whole seed (15–28 DAF); green, embryo (35–90 DAF); orange, endosperm (35–90 DAF); brown, seed coat (35–49 DAF)

To gain insight into the biological processes that are specific to the different modules, Gene Ontology (GO) enrichment analysis was performed. The five most significantly over-represented GO terms per module are depicted in Fig. 5 (see Additional file 3: Table S2 for the full dataset). The modules ME5 and ME1 contained genes that are mainly expressed during early seed development and for which it was not possible to dissect the different tissues because they were not well differentiated (Fig. 5a). These modules were described by GO terms associated with cell division/growth and morphogenesis, likely reflecting the end of embryogenesis. ME1 included several GO terms associated with “response to a stimulus, light signalling, signalling molecules (karrikins, ABA, GA, brassinosteroids) and “positive regulation of seed maturation”. This latter category contained seven *bZIP* transcription factors involved in developmental reprogramming, including orthologues of *bZIP44* and *bZIP53*, known to play a role in regulating endo-β-mannase genes and germination [45]. ME1 also contained ABA synthesis genes, including *NOTABILIS* and two orthologs of other *NINE-CIS-EPOXYCAROTENOID DIOXYGENASE* (*NCED)*. *NOTABILIS* and *SlNCED2* transcripts were highly induced early during seed embryogenesis, with *NOTABILIS* mostly present in the seed coat peaking at 35 DAF (Additional file 1: Fig. S5). Transcripts of *SlNCED6* increased both in the embryo and endosperm between 35 and 60 DAF. This is consistent with the Arabidopsis model where both a maternal and zygotic sources of ABA govern seed development [46]. Over-ripening (83–90 DAF) resulted in higher transcript levels in the endosperm. Both *SlABI3* genes were also found in ME1.

ME3 contained genes that were highly expressed between 15 to 42 DAF in all three tissues (Fig. 5a). The module was enriched in many GO terms associated with cell division, cell plate formation, growth and plant organ morphogenesis that are typical of embryogenesis (Additional file 3: Table S2). *SlWRI1*, which controls oil synthesis by regulating C allocation between fatty acids and sucrose in developing Arabidopsis seed (Fig. 3b, Additional file 1: Fig. S3), was present in this module, consistent with the presence of GO terms reminiscent of its function such as “response to sucrose” and “acetyl-CoA biosynthetic process from pyruvate” (Additional file 3: Table S2). This module also included the master regulator *SlFUSCA3* (Fig. 3a) and *SlDOG1–1* (Fig. 3i). Considering that the increase in seed weight parallels the expression profile of the eigengene of ME3, this suggests that ME3 is a gene module regulating lipid storage reserve deposition.

#### Seed coat-preferentially expressed gene modules

Module ME20 contained genes that were highly expressed between 15 and 35 DAF and was enriched in GO terms associated with growth, cell wall biogenesis and mucilage biosynthetic process (Fig. 5b), highlighting the expansion phase of the seed and differentiation of the seed coat layers. Likewise, ME15, characterized by genes transiently expressed with a peak at 35 DAF, was also enriched with cell wall associated GO terms and included terms related to “lignin biosynthesis process” and “defense response to insects” (Fig. 5b, Additional file 3: Table S2). This suggests that these two expression modules could represent a seed coat differentiation program that will set up the protective barrier just before the acquisition of germination capacity, thereby preventing precocious germination later during maturation and protecting the reproductive organ [12]. ME9 contained 399 genes with increased expression from 35 to 49 DAF. This module was characterized by GO terms associated with “pectin catabolism” and “abscission/senescence processes” (Fig. 5b) that include MAPKKK and several NAC transcription factors (Additional file 3: Table S2). Interestingly, ME9 contained several pectin lyase genes such as Solyc04g015530.3.1 that are implicated in fruit abscission. Its expression profile was consistent with the reported timing of the seed detachment from the fruit tissue in tomato [5]. It also contained the GO “response to ABA” (Additional file 3: Table S2) with several genes involved in sugar transport and synthesis of galactinol (*GolS2*, Solyc02g062590.3.1), the precursor of the raffinose family oligosaccharide (RFO, Additional file 3: Table S2, Additional file 1: Fig. S6).

#### Embryo-preferentially expressed gene modules

ME7, the largest embryo-specific module with genes expressed throughout the second phase of maturation, was enriched in many biological functions, including “response to ABA” (Fig. 5c) and “regulation of seed germination” (Additional file 3: Table S2), which might reflect repressing activities to avoid vivipary. ME8 contained 406 genes whose transcripts increased between 40 and 80 DAF in the embryo. It was characterized by an over-representation of four terms associated with organelle RNA editing (chloroplast and mitochondria RNA processing, C to U editing) that was represented only by genes encoding pentatricopeptide repeat proteins. Another significant term was “resolution of meiotic recombination intermediates”. A closer look at the gene list revealed seven genes encoding helicases and topoisomerases associated with DNA repair and the stability of repetitive sequences within the genome. The small ME17 characterizing late embryo maturation from 72 DAF onwards did not exhibit revealing GO terms (Additional file 3: Table S2) but we noted the presence of four orthologs of *HECATE* genes, a transcription factor with versatile functions throughout development [47] and *ABA2*, involved in ABA synthesis. ME13, containing genes that were highly expressed between 15 and 42 DAF, reflecting a transcriptional program related to embryogenesis (Fig. 5c).

#### Endosperm-preferentially expressed gene modules

The ME6 module, connected to the core ME1 and the embryo-specific part of ME3 (Fig. 4b-c) was composed of genes that were highly expressed early during development in whole seeds and in the endosperm from 15 to 42 DAF. The module was over-represented in GO terms associated with “jasmonic acid metabolism” (Fig. 5d). This GO term contained genes associated with several other phytohormones including salicylate, auxins, brassinosteroids and ABA (Additional file 3: Table S2), probably because of cross-talk between the hormone pathways, evident from the ortholog of *JASMONATE RESISTANT 1* (*JAR1*), an auxin responsive protein implicated in jasmonic acid (JA) synthesis. The GO term “somatic embryogenesis” (Fig. 5d) and “ABA activated signalling pathway” both revealed the presence of 10 orthologues of *LEC1-like.* ME6 was also enriched with GO terms associated with the regulation of fatty acid biosynthetic process, suggesting that ME6 represents an endosperm-specific expression module that is associated with late embryogenesis and seed filling. Genes in the ME18 module showed transient expression profile with a peak at 49 DAF, and were enriched in only one GO term with 5 genes related to suberin biosynthesis, very long fatty acids and cutin synthesis (Additional file 3: Table S2). This might reflect the differentiation of an epidermal barrier [48]. ME4 was a large expression module representing the late phase of maturation from 42 to 90 DAF. It was enriched with “gene silencing” (Fig. 5D). ME11 with 229 genes was enriched with genes involved in “chromatin silencing”, “histone deacetylation” (Fig. 5d), “negative regulation of RNA transcription” and “signal transduction” (Additional file 3: Table S2).

### Quantification of module-physiological trait associations

**Fig. 6:**
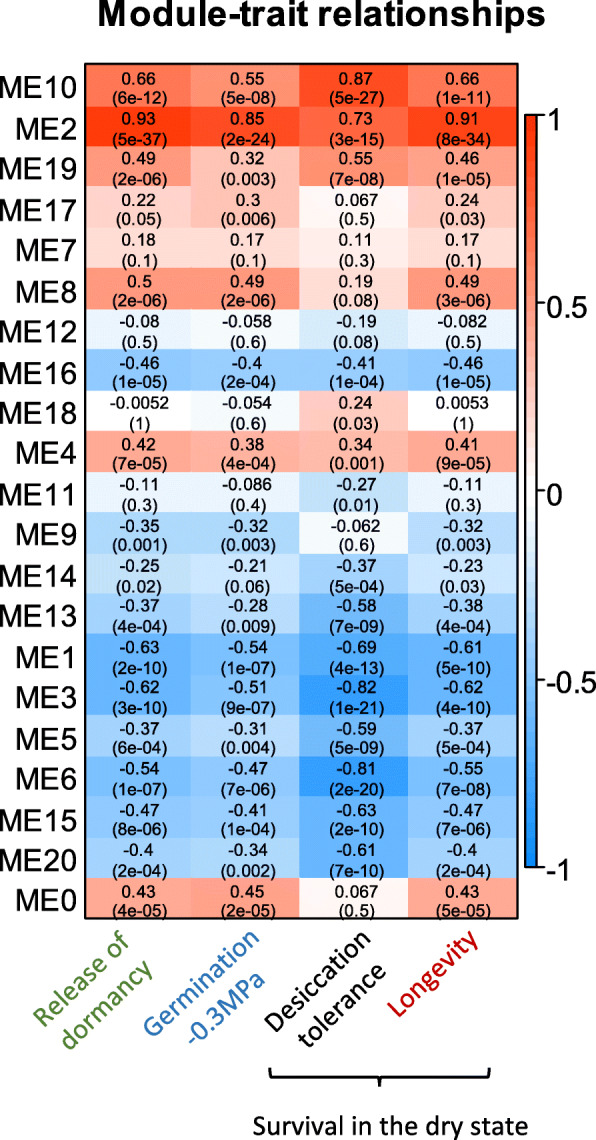
Identification of module eigengenes (ME) associated with the indicated physiological seed traits. Upper and lower value correspond to the PCC and *P*-value, respectively. The scale on the right side represents the Pearson Correlation Coefficient (PCC)

Our next objective was to incorporate the acquisition of the physiological traits (Fig. 1) into the gene network (Fig. 4b) to identify modules and hub genes that might govern these traits. To achieve this, we determined which ME modules were significantly correlated with each of the measured traits based on the Pearson correlation coefficient (PCC, Fig. 6). Acquisition of desiccation tolerance showed the highest correlation with ME10 (PCC = 0.87). This module was also correlated with P50 values, albeit with a lower level of significance. Modules ME3 (PCC = -0.82) and ME6 (PPC = -0.81) were negatively related to desiccation tolerance. Both modules were enriched in genes involved in fatty acid biosynthesis (Fig. 6, Additional file 3: Table S2). All the other vigour traits (germination/dormancy release, germination speed, germination under osmotic stress and longevity (P50) were all highly associated with module ME2. ME2 captured the late maturation phase and represented an independent subnetwork (Fig. 4). No other modules were found to be highly correlated with any of these traits (Fig. 6, Additional file 3: Table S2).

### Identification of a conserved regulatory gene module associated with desiccation tolerance

**Fig. 7:**
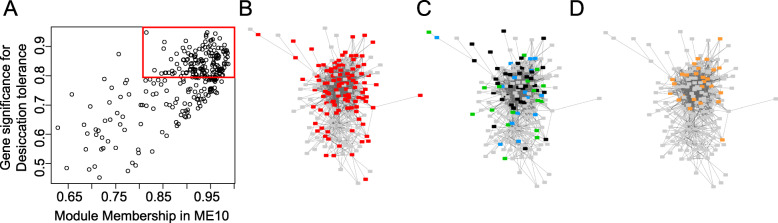
Co-expression network of ME10 revealing hub genes associated with desiccation tolerance. **a.** The relationship between gene module membership of ME10 and gene significance for desiccation tolerance (r^2^ = 0.68, *p*-value = 2.4e^− 39^). The red frame indicates highly connected genes that are highly correlated to desiccation tolerance. **b.** Topology of ME10 with seed preferentially expressed genes associated with desiccation tolerance. **c.** Topology of up-regulated genes associated with desiccation tolerance in *M. truncatula* (blue), in Arabidopsis (green), in both species (black). Data are from [49, 50], **d.** Topology of the members of the ABI3 regulon. Data are from [51]

Module ME10 was highly correlated with the acquisition of desiccation tolerance and contained 280 genes, with a large number of them being preferentially expressed in seeds (Fig. 7). Over-representation analysis revealed functions related to the “TCA cycle”, “seed oil body biogenesis”, “response to desiccation” and “response to ABA” (Additional file 3: Table S2). Since genes with high connectivity are more likely to exert large effects on physiological traits ([52] and reference therein), we identified those genes with the highest intramodular connectivity, (i.e. with the highest correlation with the eigengene of the module), as well as with the highest correlation with the acquisition of desiccation tolerance. The value of module membership (MM) of each individual gene (i.e. the PCC between the gene expression profile and the module eigengene) was plotted against the gene significance value (GS, i.e. the PCC between the expression profile of each individual gene and the seed physiological trait) (Fig. 7a). The resulting correlations between MM and GS values allowed to identify 106 hub genes having both a high MM and GS value > 0.8 (Fig. 7a, squared box, Additional file 4: Table S3). Analysis of the 25 top connected genes showed numerous genes with protective functions, namely 13 Late Embryogenesis Abundant (LEA) proteins from different families (dehydrins, D-34), one small heat shock protein (sHSP) and two oleosins (Table 1). To investigate the conserved nature, we compared our desiccation tolerance gene module with two datasets representing genes that are activated upon acquisition of desiccation tolerance in seeds of *M. truncatula* [49] and in Arabidopsis [50]. A total of 68 transcripts of the ME10 module were present in at least one of these datasets (Additional file 5: Table S4), and were clustered in the core of ME10 (Fig. 7c). They were mostly associated with protective, detoxification and repair functions. The presence of these genes across species shows that they represent a highly conserved regulatory network governing desiccation tolerance. The conserved network also contained several known regulators that are important for seed vigour (Additional file 5: Table S4), such as *SOMNUS*, a CCCH-type zinc finger transcription known to negatively regulate seed germination by activating ABA biosynthesis and inhibiting GA biosynthesis downstream of phytochrome [53], *Heat Shock Factor A2* (*HSFA2*) and two genes involved in phospholipid signalling (including *FLT/TERMINAL FLOWER 1* an ortholog of *MOTHER OF FLOWERING TIME* that is regulated by the ABA signalling pathway (Table 1, Additional file 5: Table S4). Fifty-nine percent of genes (22 genes) belonging to this conserved desiccation tolerance network represented experimentally validated direct targets of ABI3 in Arabidopsis [51]. They were tightly connected within ME10 (Fig. 7d). Outside the ABI3 regulon, additional transcription factors (TF) and several ABA signalling/responsive proteins were found associated with the desiccation tolerance network such as an ortholog of an *ABSCISIC ACID-INSENSITIVE 5-like protein 4* and a *HVA22-like protein*. (Additional file 5: Table S4).

**Table 1:** Top 25 hub genes of ME10 with transcripts that are highly connected with the module eigengene and highly correlated with the acquisition of desiccation tolerance

Gene ID	Description	GS.DT	p.GS.DT	MM.ME10	p.MM.ME10
*Solyc02g077980.3.1*	Unknown protein	0.840	1.7E-23	0.987	9.5E-67
*Solyc09g082110.4.1*	Late embryogenesis abundant protein D-34	0.878	5.1E-28	0.986	3.6E-65
*Solyc10g078780.2.1*	11 kDa late embryogenesis abundant protein	0.864	4.1E-26	0.986	4.0E-65
*Solyc07g066400.1.1*	seed maturation protein	0.887	3.2E-29	0.984	4.2E-63
*Solyc03g115370.3.1*	Diacylglycerol kinase	0.883	1.2E-28	0.984	7.0E-63
*Solyc11g042800.2.1*	Embryonic protein DC-8	0.842	1.1E-23	0.984	8.2E-63
*Solyc02g079290.3.1*	FLT/ TERMINAL FLOWER 1-like protein	0.857	2.4E-25	0.982	3.1E-61
*Solyc04g072250.4.1*	17.5 kDa class I heat shock protein	0.873	2.8E-27	0.981	2.7E-60
*Solyc09g082100.3.1*	Late embryogenesis abundant protein D-34	0.889	1.6E-29	0.980	3.7E-59
*Solyc12g098900.2.1*	Late embryogenesis abundant protein D-29	0.905	3.8E-32	0.980	4.1E-59
*Solyc03g025810.4.1*	Low-temperature-induced 65 kDa protein	0.839	2.0E-23	0.979	4.4E-58
*Solyc07g065990.1.1*	Oleosin S1–2-like	0.828	2.5E-22	0.979	4.7E-58
*Solyc09g008770.3.1*	Late embryogenesis abundant protein	0.842	1.0E-23	0.978	6.7E-58
*Solyc01g098850.3.1*	D(P)-binding Rossmann-fold superfamily protein	0.882	1.8E-28	0.977	5.1E-57
*Solyc02g084840.3.1*	Dehydrin	0.885	5.6E-29	0.977	1.1E-56
*Solyc02g091390.3.1*	Cold-regulated protein	0.819	1.8E-21	0.975	1.2E-55
*Solyc07g062990.2.1*	Late embryogenesis abundant protein 1-like	0.803	4.4E-20	0.975	1.3E-55
*Solyc01g060070.3.1*	Outer envelope pore protein 16–2, chloroplastic	0.824	6.3E-22	0.974	7.3E-55
*Solyc03g113510.2.1*	Hypothetical protein	0.817	2.7E-21	0.973	4.1E-54
*Solyc12g010820.2.1*	Late embryogenesis abundant protein	0.815	3.9E-21	0.973	7.2E-54
*Solyc02g062770.2.1*	Late embryogenesis abundant protein	0.894	2.5E-30	0.973	1.2E-53
*Solyc09g015070.3.1*	D(P)-linked oxidoreductase superfamily protein	0.821	1.1E-21	0.971	1.2E-52
*Solyc02g071760.4.1*	Oil body-associated protein 2A-like	0.809	1.3E-20	0.969	7.8E-52
*Solyc12g008430.3.1*	Malic enzyme	0.928	7.1E-37	0.969	1.1E-51
*Solyc12g038160.2.1*	Lipase	0.816	3.1E-21	0.969	1.5E-51

*GS. DT* Gene significance value associated with desiccation tolerance, *p.GS.DT* p-value of the correlation, *MM.ME10*, module membership with ME10, *p.MM.ME10 p*-value of the correlation

### Regulatory networks associated with the acquisition of seed vigour

**Fig. 8:**
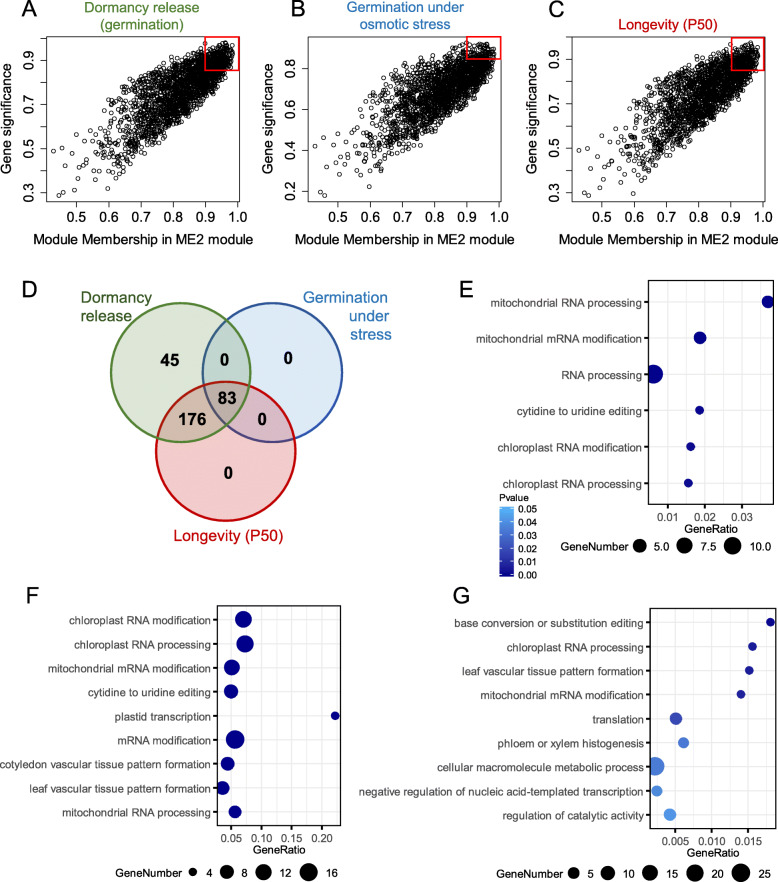
ME2 hub genes associated with seed vigour. **a.-c.** The relationship between gene module membership of ME2 and gene significance for germination (A, r^2^ = 0.8; *P* < 1e^− 200^), germination − 0.3 MPa (B, r^2^ = 0.76, *p*-value<1e^− 200^), and longevity (C, r^2^ = 0.78, *p*-value<1e^− 200^). The red frame indicates highly connected genes that are highly correlated to the indicated trait. **d.** Venn diagram of 448 top connected genes mostly correlated with dormancy release, germination under stress, and longevity. e-**g**. Major overrepresented biological functions specific to dormancy release **e**, common to dormancy release and longevity **f**, common to dormancy release, germination under stress and longevity or **g**

A similar approach as for the desiccation tolerance module ME10 was used to infer the hub genes of ME2, the module that was correlated with the dormancy release, germination under osmotic stress and longevity (Fig. 8). First, the highly connected genes with a MM > 0.9 (448 genes) were selected from the plot between MM and GS of each trait (Fig. 8a-c). A Venn diagram shows the overlap between these gene lists and revealed genes that were more correlated to a specific trait (Fig. 8 d). No hub gene was found specifically associated with germination under stress or longevity, whereas 45 genes were found exclusively for dormancy release (Fig. 8g). Over-representation analysis of the 83 common hub genes revealed an enrichment of GO terms related to “translation” and “mRNA processing/modification both in chloroplast and mitochondria” (Additional file 6: Table S5). Many genes encoding penta- and tetratricopeptide repeat containing protein were detected. Among the top correlated genes, we also found an ortholog of the SHK1 kinase binding protein1 and protein arginine methyltransferases (*PRMT5, Solyc08g005970.3.1*), a gene known to modulate pre-mRNA splicing, seed development and stress response ([54] and reference therein) and mRNA adenosine methylase (*Solyc08g066730*). The list of the 176 common genes between dormancy release and longevity was significantly enriched with mRNA processing terms also associated with the chloroplast and mitochondria (Fig. 8f, Additional file 6: Table S5). The same observation was made for the genes only correlated with dormancy. From this analysis, ME2 appeared to represent a subnetwork of regulators that couples the acquisition of seed vigour with the induction of post-transcriptional regulation both in the embryo and endosperm. Also with the genes correlating with dormancy and longevity, we detected several homologues of genes connecting light, circadian rhythm and control of flowering, such as *PHYTOCHROME AND FLOWERING TIME 1* (*PFT1*, *Solyc05g009710.4.1*), *EMBRYO DEFECTIVE 1507* (*Solyc06g065300.4.1*, a dead helicase implicated in the splicing of *FLOWERING LOCUS C* (*FLC*) [55] and *EARLY FLOWERING 4, ELF4* (*Solyc06g076960.2.1*) that synchronizes the circadian clock with light and temperature [56], an homologue of *FRIGIDA* (*Solyc04g072200.3.1*) and *PRMT5* mentioned above.

### Identification of tissue-specific modules correlating with physiological traits

**Fig. 9:**
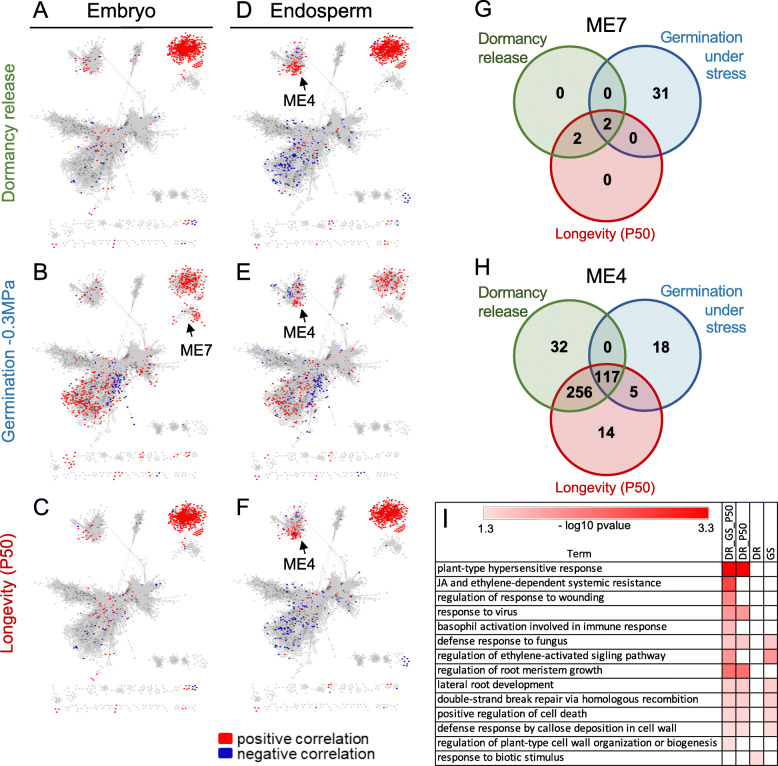
Tissue-specific hub genes associated with seed vigour. **a-f.** Distribution of positively (PCC > 0.8, red) and negatively (PCC < -0.8, blue) embryo- and endosperm-specific genes correlated with the indicated seed vigour traits on the gene network. The black arrows point to the tissue-specific subnetworks associated with seed vigour. **g-h.** Venn diagram of positively correlated genes with dormancy release, germination at − 0.3 MPa, and longevity in ME7 **g** and ME4 **h**. **i.** Comparison of most significant over-represented biological functions corresponding the different gene sets from Venn diagram **h** of ME4

So far, the approach taken to identify modules that correlated with seed vigour identified only those for which the module eigengene profile correlated with an increase in the acquisition of seed vigour both in the endosperm and embryo (Additional file 1: Fig. S7). However, this analysis excluded modules containing genes that were only expressed in either endosperm or embryo, because the correlation would have been highly correlated in only one of the seed tissues, thereby decreasing the overall PCC value with the trait throughout all the samples (Additional file 1: Fig. S7). To identify embryo- and endosperm-specific gene modules associated to seed vigour, we retained those genes whose transcript level correlated with the acquisition of seed vigour traits either in the 11 samples of the endosperm tissue or in the 11 samples of the embryo tissue. Projection of these highly correlated genes in the embryo (Fig. 9a-c) or endosperm (Fig. 9d-f) on the network highlighted as expected, the previously identified ME2 for both tissues (− 0.8 > PPC > 0.8, Fig. 9a). Comparison of the highlighted modules in Fig. 9 with the tissue-specific modules shown in Fig. 4b identified in addition one embryo-specific module (ME7, see arrow Fig. 9b), and one endosperm-specific module (ME4, see arrow Fig. 9d).

The embryo-specific ME7 contained mostly genes that were highly correlated with germination under osmotic stress (Fig. 9b). A Venn diagram depicting the genes correlating with the different traits in the ME7 module that is depicted in the network shows that only two genes (*Solyc03g095977.1.1* and *Solyc03g095973.1.*1) were found in common between the three vigour traits (Fig. 9g). Both were identified as paralogs of *ABI4*, a versatile transcription factor known to regulate dormancy in Arabidopsis [57]. The two genes that were in common between dormancy release and longevity encoded a GDSL-type esterase/lipase and *AINTEGUMENTA-like 5* (*AIL5*), also known as *CHOTTO1*. Among the 31 genes only correlated with germination under stress, three TFs and two enzymes point to a role of cell wall such as orthologues of *BEL1-Like HOMEODOMAIN 4* (*SlBLH4*, *Solyc02g065490.4.1*) and *BLH2* (Solyc04g079830.2.1), both implicated in the regulation of pectin demethylesterification, a homologue of *TRICHOME BIREFRINGENCE Like 37* (*SlTBL39*, *Solyc03g006220.4.1*), a UDP-glucosyl transferase (*Solyc05g053120.1.1*) implicated in lignin metabolism and a member of lipid-transfer protein family (*Solyc10g075050.2.1*, Additional file 2: Table S1). Also, genes implicated in the regulation of cellular growth and organellar cell identity were represented as shown by the presence of a homologue of *BLH8* (*Solyc11g069890.3.1*), *GROWTH-REGULATING FACTOR 5* (*Solyc07g041640.3.1*), *NAC DOMAIN CONTAINING PROTEIN 33* (*Solyc12g017400.3.1*) and *KANADI2* (*Solyc06g066340.4.1*).

The endosperm-specific module ME4 contained many genes that correlated strongly with dormancy release and longevity, and to a lesser extend with germination under osmotic stress (arrow Fig. 9d-f). Most of the positively correlated genes in this module (442) were found for all three traits (26%, 117 genes) or correlated with dormancy release and longevity (58%, 256 genes). A GO enrichment analysis revealed functional overlap between these two datasets (Fig. 9i, Additional file 7: Table S6). Both of them exhibited several terms broadly associated with defence response against pathogens. A more detailed look at the genes present in these gene lists identified several paralogs encoding different transposases, a DDE-4 domain-containing protein that is also closely associated with transposase activity and a SNF2 helicase, associated with DNA repair. Six paralogs encoding a serine/threonine-protein phosphatase 7 long form-like protein were found in common between germination and longevity and seven of them common to the three traits. This gene (*aka MAINTENANCE OF MERISTEMS LIKE-3, MAIL3*) participates in transposable element silencing [58]. Additional genes associated with DNA repair (*Solyc01g105520.3.1*, *Solyc01g105530.3.1*, *Solyc01g109460.3.1*) were found among genes correlating with the three traits. This suggests that a process related to genome stability is actively initiated. Next, both data sets contained genes with signalling function associated with dormancy, namely an OPDA reductase (*Solyc11g032230.3.1*), GA signalling (*GAMYB*, three paralogs of *RGA-like 3*, a GA signalling repressor inhibiting testa rupture) and two *ETHYLENE INSENSTIVE 3* (Additional file 7, Table S6). The common lists also showed many genes associated with cell wall activity, including 14 UDP-glycosyl transferases. The list of genes specifically correlated with the release of dormancy contained many genes with unknown function (Additional file 7: Table S6). A closer look at the 14 genes correlated specifically with longevity revealed orthologs of known regulators of ABA/GA signalling pathways such as *PYL5*, an ABA receptor and *RAV1* (Related to ABI3/VIP1), a B3 transcription factor modulating the expression of *ABI3*, *ABI4*, and *ABI5* [59].

## Discussion

### Late seed maturation defines a transcriptional program associated with seed vigour

Late seed maturation corresponds to the period of maturation during which seed filling is mostly terminated and seed vigour traits are acquired [9]. Within the tomato co-expression network, the expression module ME2, the endosperm-specific ME4 and the embryo-specific ME7 captured this period. Our PCA analysis of transcriptional changes together with the topology of gene modules, the expression profiles of marker genes and acquisition of vigour traits revealed that the onset of this phase starts at around 49 DAF. This also represents the moment when it became impossible to extract intact RNA from the seed coat, suggesting that its demise would be part of the late maturation program in tomato. In relation to fruit development, late seed maturation starts at least 10 days before the onset of fruit ripening or breaker stage.

Throughout late seed maturation, the seed moisture content remained high at around 50%, unlike seed development described so far at the molecular level in other species. Therefore, mechanisms involving both ABA synthesis and a low water potential within the embryo and around the developing seeds are thought to be necessary to avoid vivipary [10, 12]. In tomato, ABA content increases in the various seed tissues at around 30 days of development [10] and consistent with this, many ABA synthesis genes were found in ME1. Increasing strength of the seed coat might restrict embryonic growth [12, 60] and evidence for this was found in the seed coat specific ME15. In addition, the GO enrichment analysis of all expression modules also points to an important role of late maturation ME7 and ME12 modules in repressing vivipary and highlights putative candidate genes. Both modules are enriched in “negative regulation of germination” (GO:0010187). Within this GO term, ME12 contains hub genes corresponding to two zinc finger CCCH domain-containing proteins with respectively two and three copies. Arabidopsis seed mutants of one of the genes, *OXS2*, exhibits ABA insensitivity during germination [61]. ME7 included *SlABI4*, *SlABI5-like* and eight transcriptional regulators that are connected to, or essential for the establishment of the root quiescent center and auxin transport, including several AP2-EREP (e.g. *AIL5*, *BABYBOOM*, *PLETHORA2, SCARECROW*) and two *BHLH* transcription factors (*PHYTOCHROME INTERACTING FACTOR 8, (PIF8)* suppressing germination in Arabidopsis [62] and *ABNORMALROOT5* (ABS5) regulating vascular development and root apical meristem and auxin homeostasis. Therefore, the root apical meristem might be important in regulating pathways repressing vivipary via auxins and ABA.

Next to repressing germination while inducing seed vigour, the late maturation phase is also characterized by many GO terms related to post-transcriptional regulation, mRNA processing and gene silencing both in the embryo and endosperm. This was also found in *M. truncatula* [15], suggesting the need to reshuffle mRNA transcript population to keep those that will be mobilised to ensure germination [63, 64]. Considering that tomato seeds mature in a hydrated environment, this reshuffling is apparently not induced by maturation drying. In dry Arabidopsis seeds, stored mRNAs are conserved in ribonucleo-complexes forming mono-and polysomes that have been identified [63]. Comparison of these stored mRNAs with the tomato data sets revealed no significant overlap with monosomes (between 0.1 and 2% according to the ME) whereas in Arabidopsis, they represented 50% of the stored transcripts. It is tempting to speculate that mRNA storage in monosomal complexes might represent a specific form associated with dry seeds. Nevertheless, genes in ME2 might be necessary to synthesize the ribonucleosomal machinery that will form monosomes upon drying after harvest or during seed dispersal. This is supported by the significant enrichment of terms such as “ribosomal subunit biogenesis” that were found only in ME2. There was a significant overlap (22%) between ME2 and the Arabidopsis transcripts associated with polysomes in dry seeds compared to other tomato expression modules (3.6% overlap). Among the 22% (125) transcripts, 27 were highly correlated with the increasing vigour, making them putative candidates genes regulating post-transcriptional processes. Half of them encode tetra/pentatricopeptide-repeat domain protein, opening a new research avenue for these poorly understood domain proteins.

### Hubs genes associated with acquisition of desiccation tolerance might also contribute to longevity

Desiccation tolerance was acquired during the seed filling phase (Fig. 1), as previously observed by De Castro et al. 2006 [6] and corresponded to the gene co-expression module ME10. The comparison of ME10 genes with those previously identified with desiccation tolerance in two non-endospermic seeds [49, 50, 65] revealed a conserved network of 37 genes encoding a wide range of functions including protection (LEA and HSP), metabolism, antioxidant (1-cys-peroxiredoxins) and signalling/transcription (e.g. SOMNUS, *Solyc07g053750.1.1*). Out this conserved network, 59% (22 genes) were associated with the ABI3 regulon, confirming that it is under the regulation of ABI3 both in the embryo and endosperm. ME10 contains not only hub genes implicated in desiccation tolerance but also several genes whose loss of function leads to decreased longevity in Arabidopsis (Additional File 5: Table S4). These genes are associated with DNA and protein repair (*PARP3*, *Solyc11g067250.3.1* [66], *MSRB1 Solyc07g062060.3.1* [67] and antioxidant mechanisms (a lipocalin *Solyc07g005210.3.1* [68], a malic enzyme *Solyc08g066360.3.1*, *Solyc12g008430.3.1* [69], reviewed in Sano et al. [70]). Another gene that is involved in longevity is *galactinol synthase 1* (*SlGolS1*), the first committed enzyme in the RFO synthesis (Additional file 1: Fig. S6A [14, 60, 71]). No other transcript associated with RFO metabolism appeared in ME10 nor in the late maturation modules ME2, ME7 or ME4. The role of RFO is probably more complex in endospermic species as suggested by the expression of profile of both *GolS* and raffinose synthase genes (Additional file 1: Fig. S6). *SlSIP1* (*Solyc07g065980.3.*1), a raffinose synthase whose loss of function leads to decreased longevity in Arabidopsis [14], was highly expressed early during seed development and in the seed coat (Additional file 1, Fig. S6). Another *GolS* (*Solyc01g079170.3.*1) and several raffinose synthase genes were also highly expressed in the seed coat, suggesting that RFO metabolism might have additional roles during seed development besides protecting cellular structures in the dry state.

Maximum longevity, measured as P50 using storage conditions at 75% RH and 35 °C, was over 80 days. According to a recent longevity survey based on ageing at 60% RH, 35 °C, such high value would place tomato seeds in the long life span category, considering that the medium P50 of this category is 50d [72]. Longevity kept increasing well after the red fruit stage, considered to be the stage corresponding to maximum seed vigour [11, 73]. This result is different from a previous study on the same cultivar, which showed that after ca. 55d of development, seeds did not exhibit a further increase in longevity during storage at 40 °C, 14% moisture [73]. Such difference could not be explained by dormancy as argued before [73] because here, stored seeds were imbibed in 30 mM KNO_3_ to release dormancy. The progressive increase in longevity was concomitant to the release in dormancy (Fig. 1). This partial release of seed dormancy was also observed by De Castro et al. 2006 [6]. In contrast to studies performed by Demir et al. 1992 [73] and Berry et al. 1991 [5], in our study seeds germinated at high percentages from 45 DAF onwards. Such discrepancies might be due to differences in the environment of the mother plant that might lead to differences in the speed at which dormancy is released. Indeed, in commercial seed production settings, germination speed doubled between 45 and 70 days after pollination when developing seeds were harvested from plants grown in a rainy season with low light, warm temperature and high humidity [13]. In comparison, germination reached a maximum value after 50 days after pollination in seeds harvested from plants grown in the winter season, with twice as much light but 10 °C lower than in the rainy season [13]. Differences might also arise from the nutritive solution as the level of nitrate applied during fruit development had a profound effect on dormancy [36, 74]. In other species, the maternal environment was also found to influence seed longevity [14, 31].

### Dormancy and longevity might be controlled by pleiotropic hub genes

The temporal increase in germination reflecting the release in dormancy was concomitant with the increase in longevity. The apparent negative correlation (Fig. 1) might reflect common regulatory processes controlled in part by pleiotropic genes rather than temporal coincidence. The existence of such genes have been reported before [22, 24, 75]. In this study, a number of genes were found whose expression was highly correlated with both seed traits, being putative candidate genes that could have a pleiotropic function governing both dormancy and longevity. In the ME2 module, the presence of genes controlling flowering concurs with the concept of environmentally controlled pleiotropic role of flowering in dormancy [76]. Also we found *DORMANCY MAKER 1* (*DRM1*, *Solyc03g006360.3.1*), a auxin-regulated gene used as a marker of bud dormancy but not in Arabidopsis seed [77]. The GRAS domain of *PROCERA*, the only tomato DELLA gene, was also found to have a pleiotropic effect on dormancy and longevity [22]. *PROCERA* was not included in our network because the variability of its expression made it just below the threshold level to be retained in our analysis. However, *PROCERA* expression is consistent with a role as a putative regulator in both longevity and dormancy (Fig. 3j). The significance of a higher level of expression in the endosperm remains to be assessed. In Arabidopsis, a negative correlation between dormancy and longevity was found in mature seeds from 6 RIL populations, with GAAS5/DOG1 loci controlling both traits [24, 78]. In tomato, two homologues were found with different expression profiles. *SlDOG1–2* transcripts (*Solyc03g006120.4.1*) belonging to ME10 and expressed both in embryo and endosperm correlated negatively with the release of dormancy and increase in longevity (Fig. 3h), making it another potential candidate controlling both traits. Phylogenetic analysis of the DOG gene family in tomato is however necessary to decipher the role of DOG1 in dormancy because this gene was orthologous of *DOG-LIKE 3.*

### *ABI4* and *CHOTTO1* could be associated with the acquisition of seed vigour in the embryo

Our WGNCA analysis unveils sets of additional candidate hub genes that regulate seed vigour at the tissue level. In the embryo, three interconnected genes were found: two *SlABI4* genes and *CHOTTO1*. This is consistent with previous data showing that both genes are regulators of seed dormancy downstream of ABA and act in the same genetic pathway [57, 79], although *CHOTTO1* was reported to have a prominent role in repressing germination during imbibition and not during seed development. Since tomato seeds remain hydrated, a similar mechanism could be operating during tomato seed maturation. *ABI4* might be a gene that is more important during maturation than apparent from the literature because it was also identified in a maturation network associated with seed longevity in *M. truncatula* [15]. The precise concerted role of ABI4 and CHOTTO1 in regulating the release in dormancy and increase in longevity and why they act only in the developing embryo remains to be established. Putative leads could be that both genes constitute a cross-talk with GA [57, 79] and participate to glucose and nitrate response during germination, two metabolites regulating dormancy [80].

### The endosperm specific transcriptomic signature highlights diverse regulatory functions associated with seed vigour

In contrast to the embryo, the endosperm exhibits a large number of hub genes associated with seed vigour (Fig. 9h). Eighty-one percent of the ME4 genes were represented in the network compared to 18% of the embryo-specific ME7, suggesting a tighter co-regulation in the endosperm. The lack of connection between ME7 and ME4 in the network reinforces the idea of specific developmental trajectories with each tissues. The number and variety of GO terms associated with hormones (ABA, JA, ethylene, karrikins) suggest an important role of the endosperm in regulating seed vigour. Consistent with the high expression of *PROCERA* in the endosperm, the presence of additional genes involved in GA signalling pathway might be of significance to control seed vigour. Next to an enrichment of genes associated with defence (Fig. 9i), another intriguing observation that deserves further investigation is the significant enrichment of “gene silencing” GO term (Additional file 3: Table S2). It contains genes associated with the RNA-directed DNA methylation pathway and post-translational regulation of proteins, mechanisms known to regulate dormancy [81]. That the endosperm participates to the development of the embryo and to dormancy during imbibition is not new, but the fact that this role extends to late seed maturation and involves also longevity and germination under stress is intriguing. Altogether, this makes the tomato endosperm an excellent model to study how tissue interactions control a complex trait such as seed vigour.

## Conclusion

We constructed a high-resolution transcriptome atlas associated with the acquisition of seed vigour during maturation, using time series samples of embryo, endosperm, seed coat and whole seed, connecting seed age and fruit ripening stages. This WGCNA revealed temporal and spatial gene expression modules and identified a late maturation phase characterized by post-transcriptional regulatory processes. During this period, release of dormancy and acquisition of longevity and germination under stress are acquired concomitantly. The transcriptome reprogramming associated with desiccation tolerance appeared to be common between endosperm and embryo and partially conserved with other non-endospermic species. Co-expression analysis and gene vigour-trait based measure highlighted common and specific developmental trajectories in the embryo and endosperm that were correlated with dormancy, longevity and germination under stress. Among hub genes regulating seed vigour, ABI4 was specific to the embryo whereas genes associated with genome stability, defence against pathogens and ABA/GA signalling were specific to the endosperm. The list of candidate genes putatively controlling seed vigour point to a role of mRNA metabolism and post-transcriptional processes and flowering genes. Our data and co expression network serve as a valuable resource for the in-depth understanding of the dynamics of gene expression associated with the acquisition of seed vigour. This atlas can be data-mined and visualized using the eFP browser at BAR U Toronto at http://bar.utoronto.ca/efp_tomato/cgi-bin/efpWeb.cgi?dataSource=SEED_Lab_Angers.

## Methods

### Plant material

Seeds of *Solanum lycopersicum* cv. Moneymaker were obtained commercially from Ferme de Sainte Marthe, Angers, France. Plants were grown in 2017 and 2018 under controlled greenhouse conditions between November and May in Angers, France in 10 L pots containing substrate (Irish peat, perlite, coconut fiber; 50/40/10; v/v/v), watered with a nutrient solution and supplemented with 16 h of 250 μmol m^2^ s^− 1^ light. The day and night temperatures were maintained at > 23 °C/20 °C. Flowers were tagged daily at anthesis and developing fruits were harvested according to firstly their age from 15 to 49 DAF then their ripening stage from Breaker to Red Ripe, based on the hue colour at the equatorial and stylar regions (Additional file 1: Fig. S8). Hue colour was monitored using a colorimeter (CM-2600d, Konica Minolta, Tokyo). Mature green fruits were selected according to their position on the truss (i.e. the second fruit after the fruit at Breaker stage). Tomato seed vigour can vary according to the fruit position on the mother plant [82]. Therefore, for each developmental stage, 21–30 fruits from the 3rd to 6th trusses with first proximal and the 3 last distal fruits being discarded were harvested. For physiological analyses, seeds from at least 21 fruits from minimum 18 plants were immediately extracted by incubating the locular tissues for 1 h in pectolytic enzymes solution (Lafazym CL®, Laffort, France) followed by extensive washing with water to remove the remnants of fruit tissues. Thereafter, seeds were rapidly dried at 43% RH under airflow at room temperature then hermetically stored at 4 °C prior to seed phenotyping. For the transcriptomic analyses, seeds were manually extracted from the equatorial section of the fruit. The embryo, endosperm and seed coat were hand-dissected, immediately frozen in liquid nitrogen and stored at − 80 °C prior to RNA isolation.

### Seed trait phenotyping

Water content was determined gravimetrically on 3 to 5 replicates of 3 seeds by determination of fresh weight and dry weight after 2 days drying at 96 °C. To assess germination capacity, triplicates of 50 dried seeds were imbibed on filter paper (Whatman No1) in 9 cm diameter Petri dishes at 20 °C in the dark for 8 days. To assess germination under stressful conditions, dried seeds were imbibed in a polyethylene glycol (PEG 8000, Sigma) solution at − 0.3 MPa for 15 days [2]. Germination was scored daily as seeds exhibiting a protruded radicle of > 2 mm. To assess desiccation tolerance, seeds were imbibed at room temperature in 30 mM KNO_3_ and stratified at 4 °C for 5d to remove residual dormancy, then placed at 20 °C as mentioned above to assess germination. To test longevity, seeds were equilibrated at 75% RH for 7 d at 20 °C and then transferred to 35 °C in hermetically sealed bags. At various intervals of storage, 50 seeds were retrieved and imbibed using the desiccation tolerance protocol. Longevity was assessed as the storage time required for the seed batch to lose 50% of germination (P50) from the fit of a three-parameter log-logistic model using R.

### RNA sequencing and transcriptome analysis

Total RNA was extracted from 10 to 20 freshly harvested seeds in three biological replicates using the NucleoSpin® RNA Plant and Fungi kit (Macherey-Nagel, Düren, Germany), according to the manufacturer instructions (protocol 5.1) without the incubation step at 56 °C using the following recommended sample type: alfalfa for embryo, potato tuber for the endosperm and grape vine leaf for seed coat. RNA quantity was measured using a NanoDrop ND-1000 (NanoDrop Technologies) and quality (RIN > 7.1) was assessed using a 2100 Bioanalyzer (Agilent Technologies, Santa Clara, CA, USA). cDNA library preparation and single-end sequencing (SE50, 20 M) were conducted by Beijing Genomics Institute (https://www.bgi.com) using the DNBseq™ technology. After quality control, high quality reads were mapped on tomato genome reference SL4.0 [83] with gene models from ITAG 4.0 using quasi-mapping alignment of SALMON, version 0.14.1 [84]. For gene expression analysis, raw RNA-Seq data were first normalised as counts per million (CPM) using cpm function in edgeR package [85]. Transcripts with an average above 1 CPM in at least one developmental stage/tissue and with a coefficient of variation of log2 CPM > 0.2 among all sample types (tissues types and developmental stages) were retained for further analysis, resulting in 15,173 genes. Transcripts that were at least 10-fold more abundant in whole seeds and seed tissues compared to fruit, leaves, root and flower transcriptomes [26, 43, 44] were considered as “seed preferentially expressed”. Likewise, genes were defined as preferentially expressed in specific seed tissues if they were at least 10 times more expressed than in any other seed tissues. Differentially expressed genes (DEGs) were determined using DESeq2 package (v1.22.2) in R [86]; genes with log_2_FC > 1 or < − 1 and BH < 5% were considered as differentially expressed. Gene annotation was assigned according to the ITAG4.0 and GO terms, which were obtained using OmicsBox (https://www.biobam.com/omicsbox/). GO enrichment analyses were performed using the topGO package (v2.34.0) in R [87] applying a Fisher’s exact test and using the weight01 method. GO terms with a *p*-value < 0.001 were considered significantly enriched.

The expression data were summarized using RPKM and databased at the BAR at the University of Toronto. An image was generated using Adobe Photoshop as described in Winter et al. 2007 [88] and an XML file was generated to associate the RNA-seq samples with regions of the image. RNA-seq data may be viewed on a gene-by-gene basis by entering the gene identifier at http://bar.utoronto.ca/efp_tomato/cgi-bin/efpWeb.cgi?dataSource=SEED_Lab_Angers

### Gene co-expression network analyses and quantification of module-physiological trait associations

Co-expression network modules and hub genes were identified using the WGCNA package (v1.68) in R [40]. The automatic one step network construction was used for module detection, the power (soft threshold) was set to 26, minModuleSize to 30, maxBlockSize to 20,000, mergeCutHeight to 0.20 and TOMType was unsigned. The edge adjacency threshold was set at 0.15 and the resulting network containing 6399 nodes and 411,826 edges was visualized using a prefuse force directed layout in Cytoscape (v 3.7.1). Modules significantly associated with seed physiological trait were identified using a Pearson correlation between eigengenes expression profiles with physiological traits.

In the correlated modules, important genes were identified using gene significance (GS) measured as Pearson correlation between individual genes and physiological seed trait and module membership (MM) measured as Pearson correlation between the module eigengene and the gene expression profile. Using the GS and MM measures, identification of genes that have a high significance for the trait of interest as well as high module membership in interesting modules were allowed. Identification of embryo or endosperm genes correlating with seed physiological trait was carried out by calculating Pearson correlation between the gene expression and the physiological traits for both tissues separately. Genes with a correlation greater than 0.8 or less than 0.8 were then projected onto the gene networks in Cytoscape.

## Supplementary Information

**Additional file 1: Figure S1**. Evolution of germination of seeds harvested at indicated stages and rapidly dried at 44% RH. **Figure S2.** Loss of viability during storage at 75% RH, 35 °C for seeds harvested at indicating fruit ripening stage. **Figure S3.** Expression profiles of key regulatory genes during seed development. **Figure S4.** Two-step procedure for WGCNA module detection. **Figure S5**. Evolution of transcript levels encoding genes involved in ABA synthesis during seed development. **Figure S6**. Evolution of transcript levels encoding of galactinol synthase and raffinose synthase during seed development. **Figure S7**. RNAseq sample dendrogram and heatmap of seed traits. **Figure S8**. Changes of hue colour during tomato fruit ripening at indicated fruit region.

**Additional file 2: Table S1**. WGNCA module gene list

**Additional file 3: Table S2.** Overrepresentation analysis of biological function in the 21 genes modules

**Additional file 4: Table S3.** Module membership and gene significance associated with desiccation tolerance, dormancy release, germination under osmotic stress and longevity (P50).

**Additional file 5: Table S4.** ME10 genes associated with desiccation tolerance and ABI3 regulon

**Additional file 6: Table S5.** Overrepresentation analysis of biological functions specific or common in top connected genes (MM > 0.9) in ME2 positively associated (GS > 0.85) with dormancy release (DR), germination under osmotic stress (G0.3MPa) and longevity (P50)

**Additional file 7: Table S6.** Overrepresentation analysis of biological functions in specific or common genes positively associated with dormancy release (DR), germination under osmotic stress (G0.3MPa) and longevity (P50) in ME4.

## Data Availability

The tomato cultivar used in this article is commercially available. Seeds were purchased at “Ferme de Sainte Marthe” according to the French and European legislation. No further permission was therefore needed. The datasets supporting the conclusions of this article are available in the NCBI Gene Expression Omnibus repository, (GSE155838 and [https://www.ncbi.nlm.nih.gov/geo/query/acc.cgi?acc=GSE155838]) and in the eFP Browser. http://bar.utoronto.ca/efp_tomato/cgi-bin/efpWeb.cgi?dataSource=SEED_Lab_Angers The datasets supporting the conclusions of this article are included within the article and its additional files. Datasets obtained from web-based sources and subsequently analysed in this study are as follows. The tomato genome reference SL4.0 is available at ftp://ftp.solgenomics.net/tomato_genome/annotation/ITAG4.0_release/. The Dataset accessions that were used to determine seed-specific expression were **PRJNA391024 (**High-resolution spatiotemporal transcriptome mapping of tomato fruit development and ripening**), PRJNA282940 (**GEO: **GSE68500,** HsfA2 controls the activity of developmentally and stress-regulated heat stress protection mechanisms in tomato male reproductive tissues**) and PRJNA307656 (**GAME9 regulates the biosynthesis of steroidal alkaloids and upstream isoprenoids in the plant mevalonate pathway **available at** https://www.ncbi.nlm.nih.gov/bioproject/

## Abbreviations

ABA: Abscisic acid
CPM: Counts per million
DAF: Days after flowering
DEG: Differentially expressed genes
DT: Desiccation tolerance
DW: Dry weight
Em: Embryo
End: Endosperm
GA: Gibberellic acid
GO: Gene ontology
GS: Gene significance
JA: Jasmonic acid
LALF: LEC1, ABI3, FUS3, and LEC2
ME: Module eigengene
MM: Module membership
PCA: Principal component analysis
PCC: Pearson’s correlation coefficient
QTL: Quantitative trait loci
RFO: Raffinose family oligosaccharide
RH: Relative humidity
RIL: Recombinant inbred line
RNA-seq: RNA sequencing
S: Seed
SC: Seed coat
TCA: Tricarboxylic acid
TF: Transcription factor
WGCNA: Weighted gene coexpression network analysis
