# Supplementary material for: Gene co-expression analysis of tomato seed maturation reveals tissue-specific regulatory networks and hubs associated with the acquisition of desiccation tolerance and seed vigour
Source: BMC Plant Biol. 2021 Mar 1;21:124. doi: 10.1186/s12870-021-02889-8 (PMC7923611; doi:10.1186/s12870-021-02889-8)
Supplement: Supplementary file 1 — Additional file 1: Figure S1. Evolution of germination of seeds harvested at indicated stages and rapidly dried at 44% RH. Figure S2. Loss of viability during storage at 75% RH, 35 °C for seeds harvested at indicating fruit ripening stage. Figure S3. Expression profiles of key regulatory genes during seed development. Figure S4. Two-step procedure for WGCNA module detection. Figure S5. Evolution of transcript levels encoding genes involved in ABA synthesis during seed development. Figure S6. Evolution of transcript levels encoding of galactinol synthase and raffinose synthase during seed development. Figure S7. RNAseq sample dendrogram and heatmap of seed traits. Figure S8. Changes of hue colour during tomato fruit ripening at indicated fruit region. [file 12870_2021_2889_MOESM1_ESM.pdf]

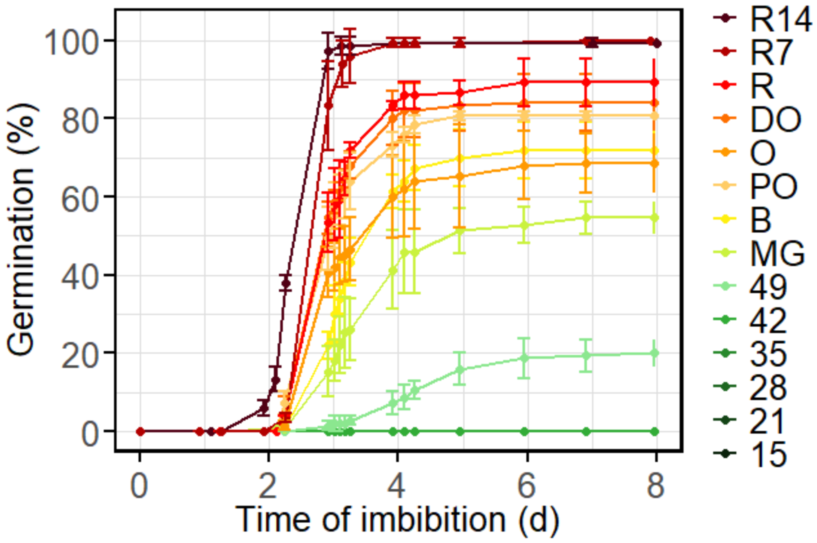

Figure S1: Evolution of germination of seeds harvested at indicated stages and rapidly dried at 44 % RH. Seeds were imbibed in water at 20°C in the dark. Data are the mean of 3 replicates of 50 seeds. 15, 21, 28, 35, 42 and 49 correspond to days after flowering. Fruit ripening stages: MG, mature green; B, breaker; PO, pale orange; O, orange; DO, dark orange; R, red; R7, red plus 7 days; R14, red plus 14 days

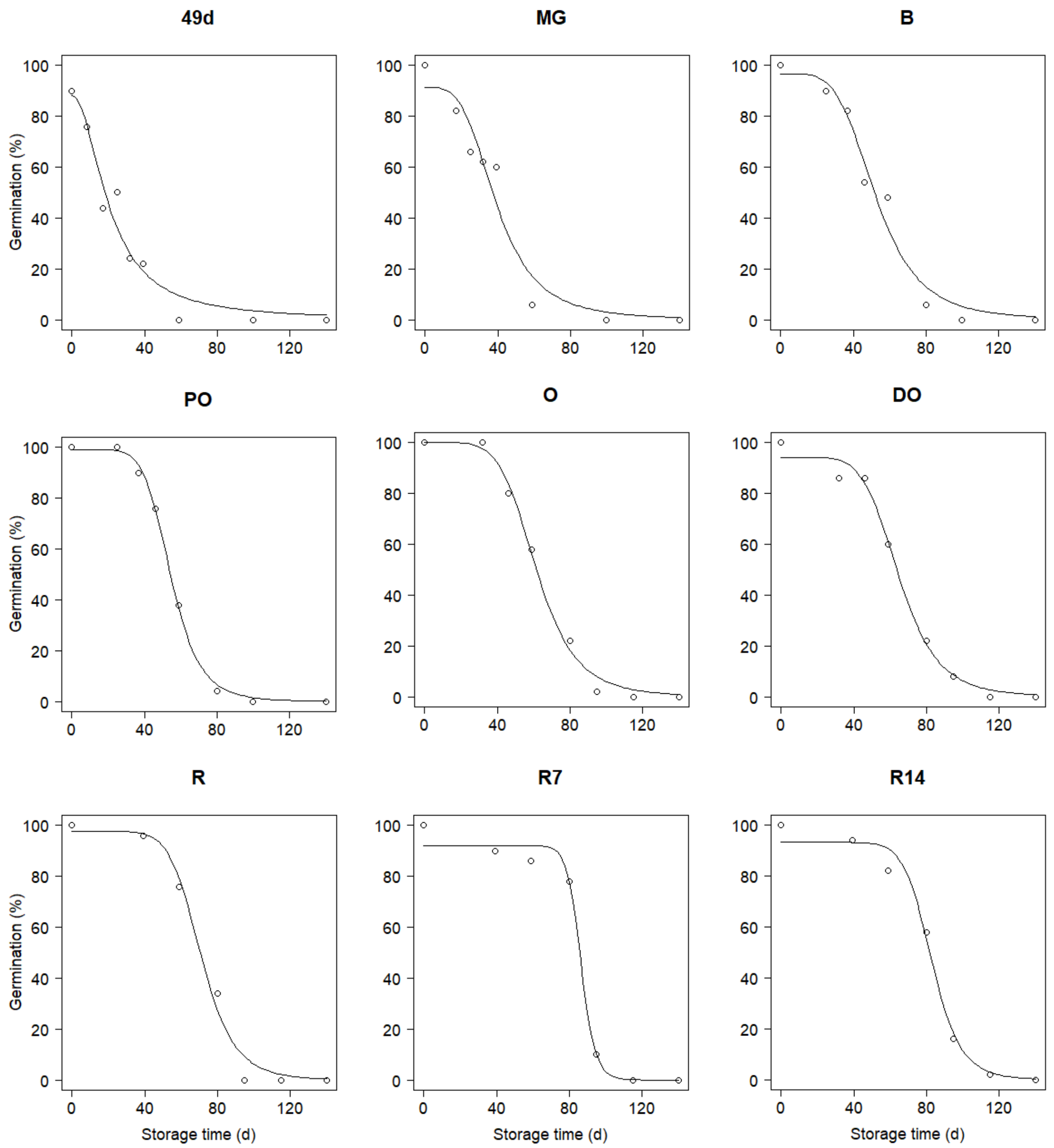

Figure S2 Loss of viability during storage at 75% RH, 35°C for seeds harvested at indicated fruit ripening stage. Viability was measured as germination at 20°C in the dark in the presence of 30 mM KNO_3. Data (n= 50 seeds) were fitted with a three-parameter log-logistic model to calculate P50, the storage period necessary to obtain 50% germination.

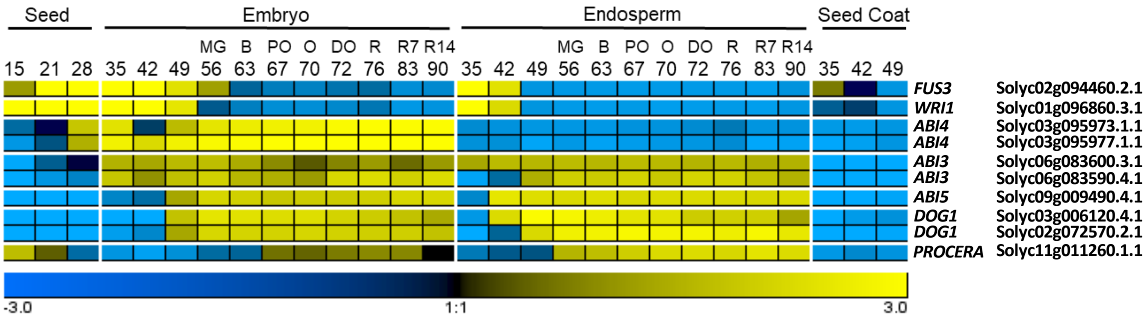

Figure S3 : Expression profiles of key regulatory genes during seed development. Seeds or seed tissues were harvested at the indicated days after flowering. The corresponding fruit ripening stage is also shown. Data represent the $\log_2$ CPM+1 value after mean centered normalization.

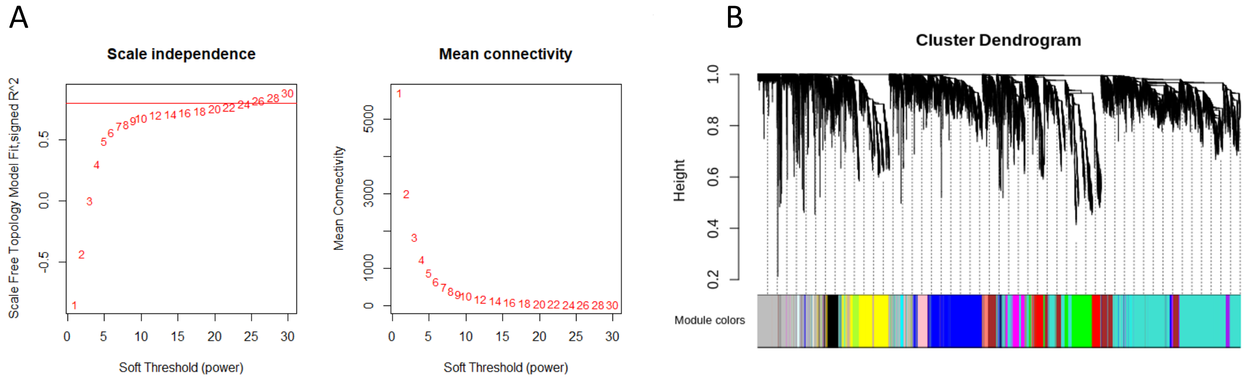

Figure S4 : Two step procedure for WGCNA module detection. **a.** Selection of the soft thresholding power with scale independence and mean connectivity. Power 26 was chosen for which the fit index curve reached a value >0.8. **b.** Cluster dendrogram and module assignment. Twenty-one modules were identified and represented in color underneath the dendogram.

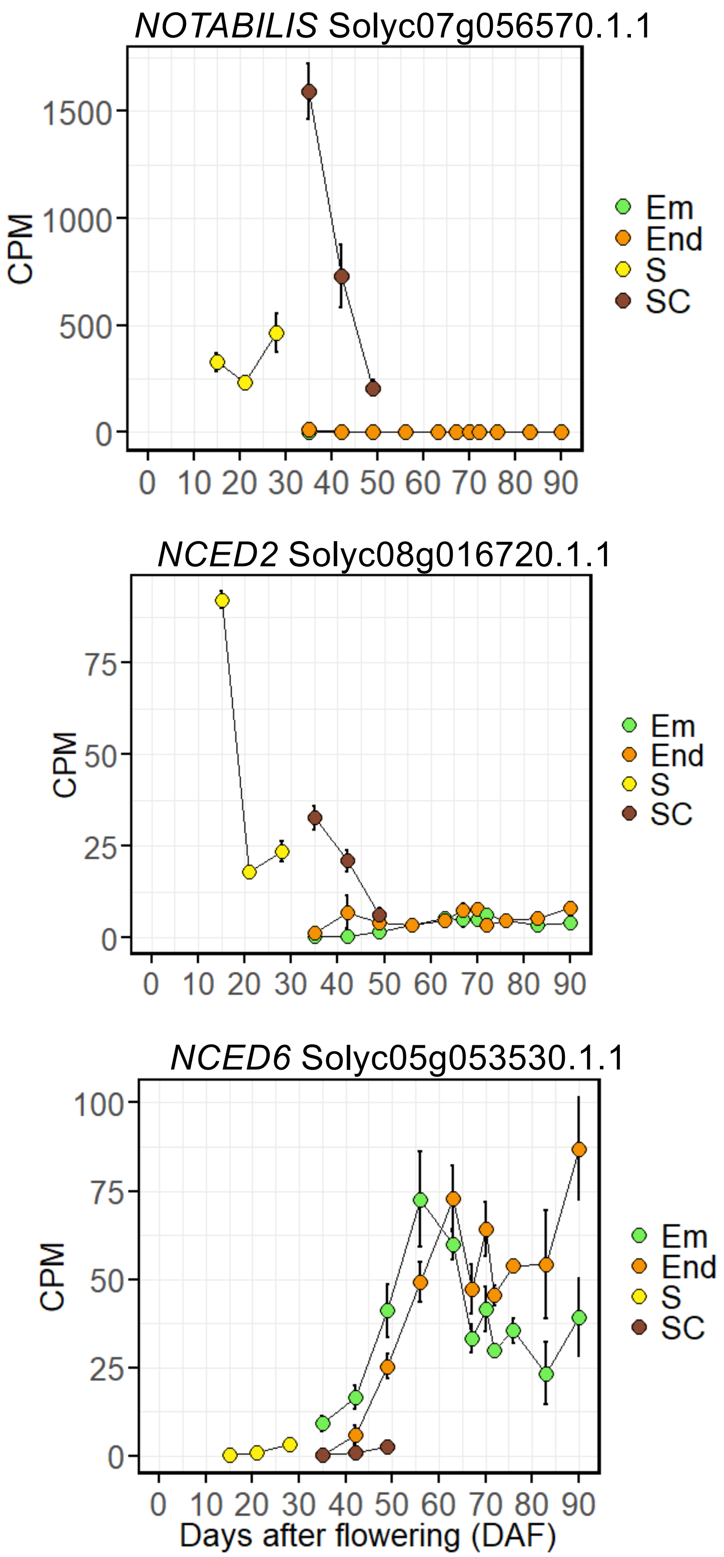

Fig. S5 Evolution of transcript levels encoding genes involved in ABA synthesis during seed development. All genes belong to ME1. Em, embryo; End, endosperm, S, whole seeds, SC, seed coat.

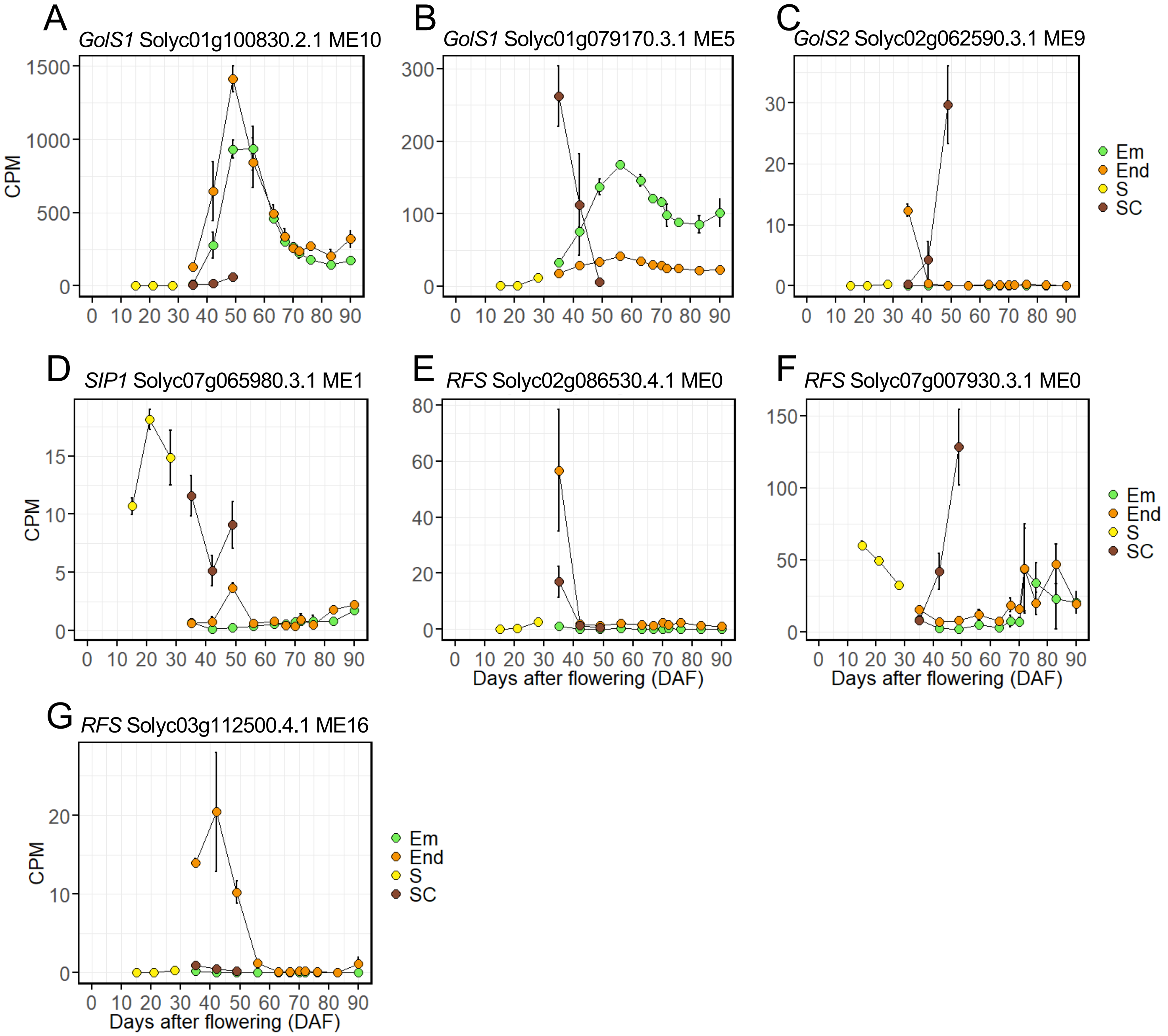

Fig. S6 Evolution of transcript levels encoding galactinol synthase (GolS) (A-C) and raffinose synthase (RFS) during seed development (D-G). Module eigengene (ME) are indicated. Em, embryo; End, endosperm, S, whole seeds, SC, seed coat.

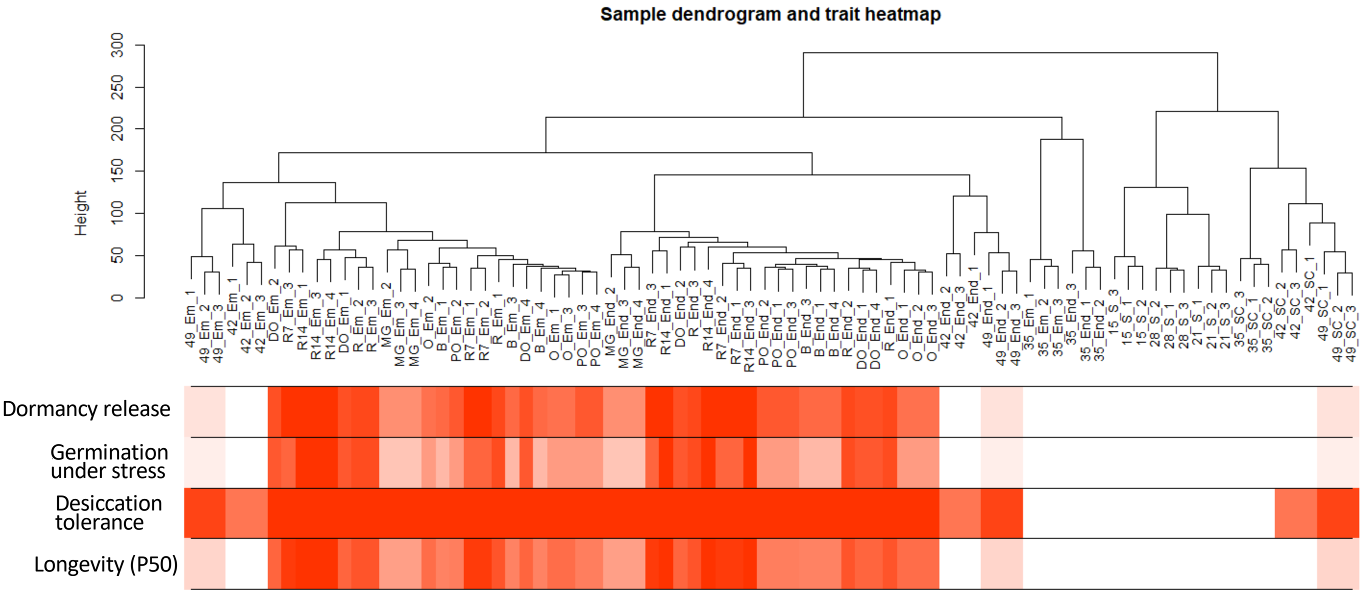

Figure S7 : RNA seq sample dendrogram and heatmap of seed traits. The clustering was based on the expression profiles. The red intensity was proportional to the different seed traits, namely dormancy release, germination under stress, desiccation tolerance and longevity. Em, embryo; End, endosperm; S, whole seed; SC, seed coat; 15, 21, 28, 35, 42 and 49 correspond to days after flowering. Fruit ripening stages: MG, mature green; B, breaker; PO, pale orange; O, orange; DO, dark orange; R, red; R7, red plus 7 days; R14, red plus 14 days

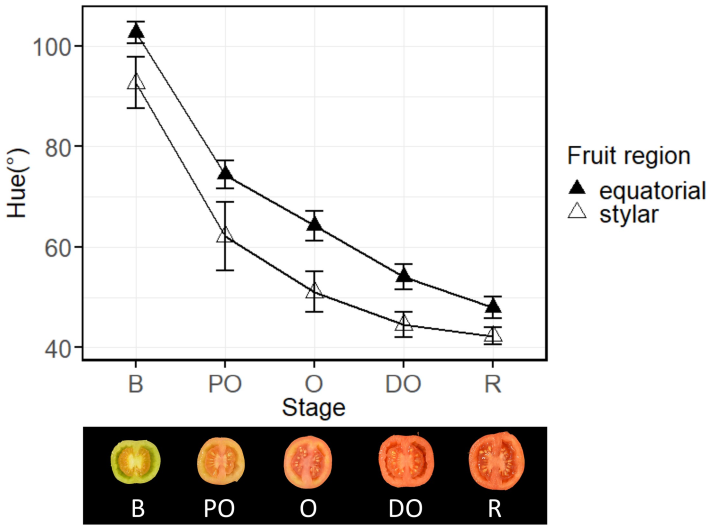

Figure S8 : Changes of hue color during tomato fruit ripening at indicated fruit region. B, breaker; PO, pale orange; O, orange; DO, dark orange; R, red.
